# Pleiotropic Impacts of Macrophage and Microglial Deficiency on Development in Rats with Targeted Mutation of the *Csf1r* Locus

**DOI:** 10.4049/jimmunol.1701783

**Published:** 2018-09-24

**Authors:** Clare Pridans, Anna Raper, Gemma M. Davis, Joana Alves, Kristin A. Sauter, Lucas Lefevre, Tim Regan, Stephen Meek, Linda Sutherland, Alison J. Thomson, Sara Clohisey, Stephen J. Bush, Rocío Rojo, Zofia M. Lisowski, Robert Wallace, Kathleen Grabert, Kyle R. Upton, Yi Ting Tsai, Deborah Brown, Lee B. Smith, Kim M. Summers, Neil A. Mabbott, Pedro Piccardo, Michael T. Cheeseman, Tom Burdon, David A. Hume

**Affiliations:** * The Roslin Institute, The University of Edinburgh, Easter Bush EH25 9RG, United Kingdom;; † The University of Edinburgh Centre for Inflammation Research, The Queen’s Medical Research Institute, Edinburgh EH16 4TJ, United Kingdom;; ‡ New World Laboratories, Laval, Quebec H7V 5B7, Canada;; § Nuffield Department of Clinical Medicine, University of Oxford, John Radcliffe Hospital, Headington, Oxford OX3 9DU, United Kingdom;; ¶ Department of Orthopaedic Surgery, The University of Edinburgh, Edinburgh EH16 4TJ, United Kingdom;; ‖ School of Chemistry and Molecular Biosciences, The University of Queensland, Brisbane, Queensland 4072, Australia;; # Medical Research Council Centre for Reproductive Health, The University of Edinburgh, Edinburgh EH16 4TJ, United Kingdom;; ** Faculty of Science, University of Newcastle, Callaghan, New South Wales 2309, Australia; and; †† Mater Research-University of Queensland, Brisbane, Queensland 4101, Australia

## Abstract

We have produced *Csf1r*-deficient rats by homologous recombination in embryonic stem cells. Consistent with the role of *Csf1r* in macrophage differentiation, there was a loss of peripheral blood monocytes, microglia in the brain, epidermal Langerhans cells, splenic marginal zone macrophages, bone-associated macrophages and osteoclasts, and peritoneal macrophages. Macrophages of splenic red pulp, liver, lung, and gut were less affected. The pleiotropic impacts of the loss of macrophages on development of multiple organ systems in rats were distinct from those reported in mice. *Csf1r^−/−^* rats survived well into adulthood with postnatal growth retardation, distinct skeletal and bone marrow abnormalities, infertility, and loss of visceral adipose tissue. Gene expression analysis in spleen revealed selective loss of transcripts associated with the marginal zone and, in brain regions, the loss of known and candidate novel microglia-associated transcripts. Despite the complete absence of microglia, there was little overt phenotype in brain, aside from reduced myelination and increased expression of dopamine receptor-associated transcripts in striatum. The results highlight the redundant and nonredundant functions of CSF1R signaling and of macrophages in development, organogenesis, and homeostasis.

## Introduction

Resident macrophages are abundant in all tissues, where they adapt to distinct niches and environments, expressing specific genes to perform tissue-specific homeostatic functions (reviewed in Ref. 1, 2). Macrophage differentiation from progenitor cells, and many aspects of their mature function, is controlled by two ligands, CSF1 and IL-34, which both signal through the CSF1R. The biology of CSF1R and its ligands is conserved from birds to mammals (3). *Csf1r* expression in adults is restricted to cells of the myeloid lineages, and transcriptional regulation of the gene has been studied extensively, both in vitro and with the use of transgenic mice, chickens, and sheep (4–6).

In humans, mutations in the tyrosine kinase domain of CSF1R have been associated with a neurodegenerative disease, autosomal dominant, adult-onset leukoencephalopathy with axonal spheroids and pigmented glia (ALSP, previously known as hereditary diffuse leukoencephalopathy with spheroids) (7). Disease-associated mutant CSF1R isoforms can be expressed on the cell surface but lack ligand-dependent kinase activity and probably act as dominant negative repressors of the wild type allele (8).

Targeted mutation of *Csf1r* in mice depleted tissue macrophages from most organs and had pleiotropic impacts on growth and development (9). The more penetrant phenotype of the receptor mutation, compared with the natural mutation of the *Csf1* ligand in the *Csf1*^op/op^ mouse, predicted the existence of the second ligand, IL-34 (9). Phenotypes of the *Csf1* or *Csf1r* mutant mice include increased bone density (osteopetrosis), abnormalities of the sensory nervous system, global defects in brain development, infertility, failure of pancreatic β cell development, and severe postnatal growth retardation (reviewed in Ref. 10, 11). On the original cross-bred background, around 50% of *Csf1r*^−/−^ mice survived to weaning. On an inbred background, survival beyond 3 weeks was rare (12).

Rat models have been used extensively in the study of human disease and are preferred to mice in areas of inflammation, development, physiology, neurobiology, pharmacology, and behavior (reviewed in Ref. 13). The use of rats has recently been accelerated by the expanding availability of genetic modification technologies via homologous recombination in embryonic stem cells (ESC) and, more recently, CRISPR–Cas9 and other targeted nucleases. This has led to a comeback of the rat in biomedical research (14). Rats provide many experimental advantages over mice such as larger sample volumes, higher resolution in vivo imaging, improved performance in learning/memory behavioral assays, easier surgical procedures, and physiological similarities with humans (15). They are the model of choice in areas such as diabetes, breast cancer, and chronic inflammatory and cardiovascular diseases as well as age-related illnesses (reviewed in Ref. 16). Current knowledge of CSF1R biology in rats has been based upon limited analysis of the *toothless* (*tl/tl*) mutation in the ligand CSF1, focused largely upon the osteopetrotic phenotype in bone (17). In this article, we report on the generation *Csf1r*^−/−^ rats by homologous recombination in ESC and their detailed phenotypic characterization. The results highlight significant differences in CSF1R between species and suggest the rat may be a better predictive model for human macrophage biology.

## Materials and Methods

### Production of Csf1r^−/−^ rats

An EGFP-PGK-Neo cassette flanked by 2.2 kb 5′ and 5 kb 3′ homology arms (Supplemental Fig. 1A) was introduced into ESC derived from a male Dark Agouti rat [clone DAK31 (18) provided by Austin Smith] by electroporation. Positive selection was provided by a PGK-Neo cassette, and negative selection was provided by a diphtheria toxin-A chain cassette. The diphtheria toxin-A chain cassette was placed upstream of the 5′ homology arm and so was not incorporated into the genome. *Csf1r* gene-targeted rat ESC clones were identified by 5′ PCR screening and 3′ Southern blot analysis. Of the 24 clones analyzed, 20 were positive at the 5′ end for the targeted allele by PCR, and 18 were positive at the 3′ end for the targeted allele by Southern blot (Supplemental Fig. 1D, 1E). Fifty percent of clones analyzed had normal karyotypes (data not shown), and clone DAK31-C2 was used to generate the colony (Supplemental Fig. 1B).

All experiments were carried out under the authority of a U.K. Home Office Project License under the regulations of the Animals (Scientific Procedures) Act 1986. Approval was obtained from ethics committees of The Roslin Institute and The University of Edinburgh. *Csf1r^−/−^* rats were produced by blastocyst injection as described in (19), except that Sprague–Dawley females were used as both embryo donors and pseudo-pregnant recipients. Genotyping and the 5′ PCR screen of ESC clones was performed by PCR using MyTaq HS DNA polymerase (Bioline) with the primers forward: 5′-GCTGCAGTCCCTTACATAGGTCTAC-3′, reverse 1: 5′-GGAGGTGCAGATGAACTTCAGG-3′, and reverse 2: 5′-AGCTTCCCCTGCCCTGAGAAG-3′. Expected products were 3.5 and 2.9 kb for the wild type and mutant *Csf1r*, respectively (Supplemental Fig. 1C).

### 3′ Southern blot analysis of ESC clones

Genomic DNA (7.5 μg) from ESC clones were digested with Xbal and used in Southern blot analysis as per standard procedures. The probe was 503 bp and spanned the third exon of rat *Csf1r* (Rat Genome Sequencing Consortium 6.0/rn6, chr18:56,437,991–56,438,493).

### Gene expression analysis

Livers and spleens were preserved in RNA*later* (Invitrogen). Total RNA was isolated in TRIzol (Invitrogen), followed by purification with an RNeasy Mini kit (QIAGEN) according to instructions. cDNA synthesis and quantitative real-time PCR (qRT-PCR) were performed as described in (6). The oligonucleotides used are listed in Table I.

### Radiographs and micro–computed tomography analysis of bone architecture

Radiographs were produced at the Hospital for Small Animals located at the Royal (Dick) School of Veterinary Studies (Easter Bush, U.K). Micro–computed tomography (μCT) analysis was performed on formalin fixed tibias and skulls using a SkyScan 1172 (Bruker) at a resolution of 17.22 μm with a 0.5-mm aluminum filter and four-frame averaging to improve the signal to noise ratio of the images. The tibias were scanned using a source voltage of 71 kV and a source current of 139 μA. To accommodate the larger dimensions of the skulls, a double width acquisition mode was used with a source voltage of 81 kV and a source current of 122 μA. An exposure time of 1180 ms was used for all scans. The images were reconstructed using NRecon v1.6.9 (Bruker) and analyzed using CTAn v1.13.5 (Bruker) software.

### Complete blood count analysis

Whole EDTA blood was analyzed as described in (20).

### Serum biochemistry analysis

Serum was analyzed using the ILab 650 automated biochemistry analyzer (Instrumentation Laboratory).

### Flow cytometry

Blood was collected into EDTA tubes via cardiac bleeds and prepared using Dako Uti-Lyse erythrocyte lysing solution according to instructions. Blood was stained with AF647-labeled CSF1-Fc (6) and the following Abs: CD32^APC^ (Clone REA256, 1:40) and CD161^APC^ (Clone REA227, 1:40) from Miltenyi Biotec, B220^PE^ (Clone His24, 1:400; eBioscience), CD4^FITC^ (Clone W3/25, 1:10; Bio-Rad Laboratories), CD3^AF488^ (Clone 1F4, 1:200), and SIRPα^PE^ (Clone OX-41, 1:400) from BioLegend. Myelin-depleted single-cell suspensions of brain were prepared as described in (21), except rats were perfused with physiological saline and heparin and then stained with CD45^EF450^ (Clone OX1, 1:200; eBioscience) and CD11b/c^AF647^ (Clone OX-42, 1:500; BioLegend). Peritoneal cavity cells were isolated as described in (22) and stained with SIRPα^PE^ and CD11b/c^AF488^ as described above. Isotype controls were used for all flow cytometry data. Cells were analyzed by flow cytometry on a FACSCalibur, LSRFortessa X-20 or LSRFortessa (BD Biosciences). Analysis was performed with FlowJo software (FlowJo).

### Western blot

Plasma was separated from EDTA whole blood by centrifugation at 2000 G for 15 min at 4°C. A 1:50 dilution was prepared using SDS-PAGE loading buffer. Proteins were separated by SDS-PAGE and transferred to nitrocellulose membranes for immunoblotting. Membranes were probed with polyclonal primary Abs: rabbit anti-CSF1 (1:500, MBS551037; MyBioSource) and 1:2000 sheep anti-Transferrin (1:2000, ab9033; Abcam) in PBS/3% milk powder/0.1% (v/v) Tween 20 at 4°C overnight. The secondary Abs used were goat anti-rabbit HRP (1:5000, no. 7074; Cell Signaling Technologies) and rabbit anti-sheep HRP (1:10,000, ab97130; Abcam). Blots were visualized using Pierce ECL Western blotting solution (Thermo Fisher Scientific).

### Histology and immunohistochemistry

Formalin fixed organs were processed into paraffin using standard procedures. For histological examination, sections were stained with H&E or Luxol fast blue. For immunohistochemical analysis, details of Ag retrieval, Ab concentrations, and detection systems used are listed in Table II. Femurs were decalcified in EDTA, and sections were stained using an acid phosphatase and tartrate-resistant acid phosphatase (TRAP) kit (Sigma-Aldrich) according to instructions, except incubation time was increased to 2 h, and a counterstain was not used. Epidermal sheets were obtained after incubating the dorsal face of both ears in 20 mM EDTA for 2 h (37°C), followed by fixation in 4% PFA for 20 min and washing in PBS. Tissues were blocked for 1 h (room temperature [RT]) in PBS/0.1% BSA/5% goat serum, then stained with anti-rat MHC class II (MHC-II) (clone OX-6, 1:100; Abcam). Secondary Ab was F(ab′)_2_-Goat anti-Mouse IgG (H+L)^AF647^ (1:500, A-21053; Invitrogen). Epidermal sheets were washed in HBSS (Thermo Fisher Scientific), and nuclei were stained for 5 min (RT) with Hoechst 33258 (1:1000, 861405; Sigma-Aldrich). Stained epidermal sheets were mounted with ProLong Gold (Life Technologies) and cured for 24 h (RT) before imaging.

### Microarrays

Total RNA was prepared from snap frozen brain regions (hippocampus, striatum, olfactory bulbs, and pituitary gland) and spleens as described above from nonperfused rats (two female rats per genotype). Library preparation and hybridization to the Affymetrix Rat Gene 2.1 ST array was performed by Edinburgh Genomics, The University of Edinburgh. CEL files were normalized [RMA (23)] and annotated in R/Bioconductor. The data from the microarrays are available at Gene Expression Omnibus, National Center for Biotechnology Information (http://www.ncbi.nlm.nih.gov/geo/), under accession number GSE100696. To identify spleen-specific genes downregulated by the loss of *Csf1r*, the normalized transcriptomic data were loaded into Graphia Pro (Kajeka, U.K.). Using a Pearson correlation threshold cutoff of R = 0.98, a graph was obtained comprising 20,515 nodes (individual probe sets). Clustering of the graph using the Markov clustering algorithm (MCL) was used with an MCL inflation value of 1.5 to determine the granularity of clusters.

### Evaluation of blood–brain barrier permeability

A 2% solution of Evans blue in PBS (4 μl/g body weight) was injected i.p. in postnatal day 17 rats. The stain was allowed to circulate for 3 h before rats were culled by cervical dislocation. Spleens and brains were removed, then weighed. The organs were dried overnight in a 55°C oven, and Evans blue was extracted by addition of formamide (16 μl/mg spleen and 8 μl/mg brain). After 48 h at 55°C, Evans blue stain was measured by spectrophotometer at 620 nm and quantified according to a standard curve. The results are presented as microgram of Evans blue per milligram of dried tissue.

### Statistical analysis

Analysis was performed with GraphPad Prism version 6.04, and statistical significance was assessed using Student two-tailed *t* test. Resulting values were considered statistically significant at *p* < 0.05.

## Results

### Generation and gross phenotype of Csf1r^−/−^ rats

**FIGURE 1.**
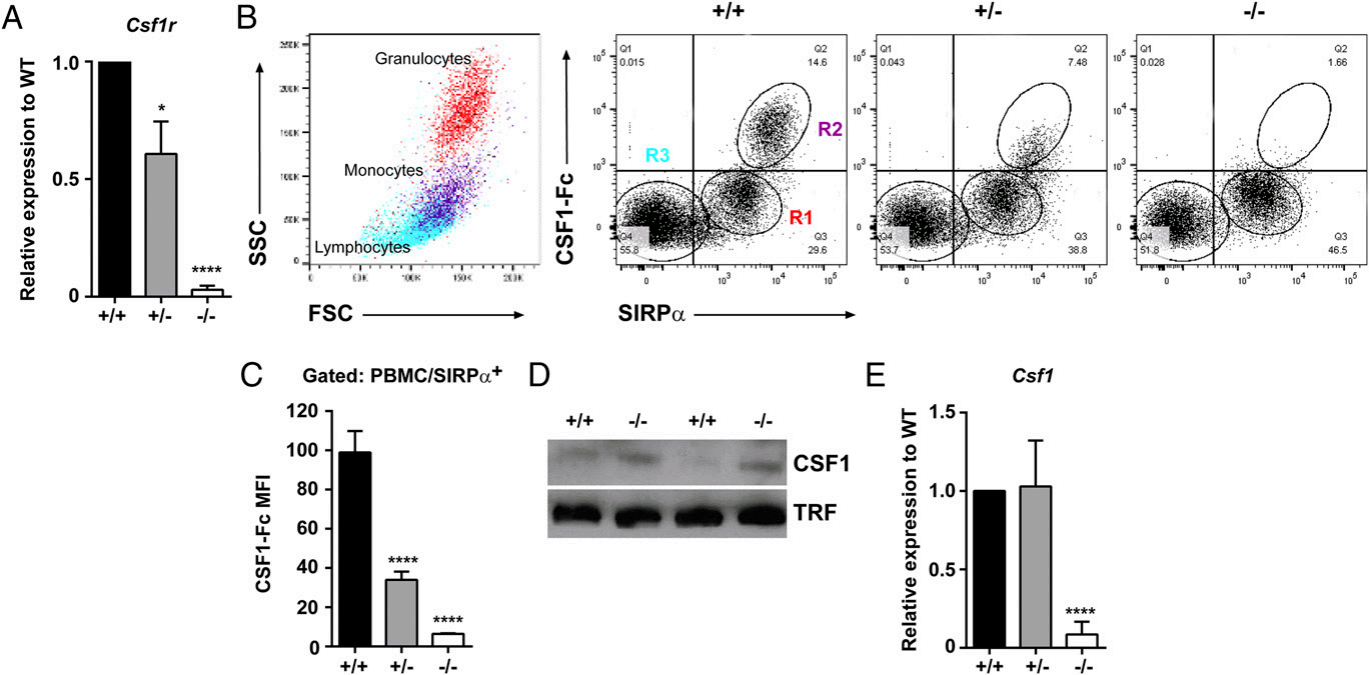
Expression of *Csf1r* and *Csf1* in rats. (**A**) cDNA was prepared from total splenic RNA and *Csf1r* expression analyzed by qRT-PCR (*n* = 7^+/+^, 7^+/−^, and 3^−/−^). (**B**) Whole EDTA blood was used to analyze SIRPα expression and binding of CSF1R to its ligand (CSF1-Fc) by flow cytometry. Regions R1–R3 highlighted in *Csf1r*^+/+^ rat blood were back-gated onto a forward versus side scatter (FSC/SSC) dot plot to highlight the three main cell populations in blood. (**C**) Combined mean fluorescent intensity of CSF1-Fc binding in SIRPα^+^ monocytes (*n* = 6^+/+^, 8^+/−^, and 5^−/−^). (**D**) Protein lysates were prepared from EDTA plasma (female adults) and assessed for CSF1 expression by Western blot. An anti-transferrin (TRF) Ab was used as the loading control. (**E**) cDNA was prepared from total spleen RNA and *Csf1* expression analyzed by qRT-PCR (*n* = 7^+/+^, 7^+/−^, and 3^−/−^). Graphs show the mean + SEM. Significance compared with wild type is indicated by *p = 0.015 and ****p < 0.0001 using a *t* test.

*Csf1r^−/−^* rats were produced using ESC in which the first coding exon of rat *Csf1r* was disrupted with a drug selection cassette by homologous recombination (Supplemental Fig. 1A). qRT-PCR demonstrated partial (∼50%) and complete loss of *Csf1r* mRNA in spleens from *Csf1r*^+/−^ and *Csf1r*^−/−^ rats, respectively (Fig. 1A), indicating that there was no dosage compensation in the heterozygote. The loss of functional CSF1R was demonstrated using a labeled CSF1R ligand [porcine CSF1-Fc (6)]. There was reduced (>60%) binding activity on monocytes (SIRPα^+^, SSC^lo^ cells) from *Csf1r*^+/−^ heterozygous rats. There was no CSF1 binding activity on any blood cells from *Csf1r*^−/−^ rats (Fig. 1B, 1C). The loss of CSF1R activity was associated with an increase in the ligand in the circulation, as observed in *Csf1r*^−/−^ mice (9), confirmed by Western blot analysis (Fig. 1D). By contrast, *Csf1r*^−/−^ rats displayed a significant reduction in *Csf1* mRNA in the spleen (Fig. 1E). This likely reflects the depletion of CSF1-dependent macrophages, which in rats (unlike mice, see data on www.biogps.org or www.immgen.org) express abundant *Csf1* mRNA (see below).

**Table I.** Oligonucleotides

Gene	Forward Oligonucleotide	Reverse Oligonucleotide
*Adgre4*	5′-TGCCCTTATTGTTGCTGTGTCTGC-3′	5′-CACTGGCCCCAAGAAGCTCCA-3′
*Cd209b*	5′-TCCAAGATCCCCAGCCTCCAG-3′	5′-GCAGAGTCGACACAGGCGGA-3′
*Csf1r* (genotyping)	5′-GCTGCAGTCCCTTACATAGGTCTAC-3′	R1, 5′-GGAGGTGCAGATGAACTTCAGG-3′
		R2, 5′-AGCTTCCCCTGCCCTGAGAAG-3′
*Csf1*	5′-AGTCTTGCTGGCTGTCGGGG-3′	5′-GGTCGCCCCACAGAAGAATCCA-3′
*Csf1r*	5′-ACGGCCACCATGAACTTCCA-3′	5′-CGCAGGGTGAGCTCAAAGGT-3′
*Fmod*	5′-CCCTCCCGTCAACACCAACCT-3′	5′-AAGTTCATGACGTCCACCACCG-3′
*Gal3st2*	5′-GCTGGCTGTGCTCCTGTTGG-3′	5′-GCGGTTCCTGGGCCTTGTCC-3′
*Gapdh*	5′-ATGACTCTACCCACGGCAAG-3′	5′-TGGGTTTCCCGTTGATGACC-3′
*Ghr*	5′-CGGGTGTTCTTAACCCTGGCACT-3′	5′-GCAGAACCGGGGAAGCTTTGC-3′
Total *Igf1**^a^*	5′-TGTGTGGACCAAGGGGCTTT-3′	5′-GTCTGTGGTGCCCTCCGAAT-3′
*Il22ra2*	5′-GGACACCCCGCTTCACTCCA-3′	5′-CCCTCAAAGATGCATTAACTCGGGT-3′
*Mpeg1*	5′-GGTTTGCCGGGTCCCTTGGT-3′	5′-ACATTCGTGCAGCCAGGGTG-3′
*Nr1h3 (Lxrα)*	5′-GCAGAGACCCTCCCAGAGCCTA-3′	5′-ACACTGCATAGCTCGTTCCCCAG-3′

^*a*^ Spans local and circulating transcripts.

R, reverse.

**Table II.** Immunohistochemistry conditions

Target (Clone)	Supplier	Ag Retrieval	Dilution	Detection
CD68 (ED1)	Bio-Rad	Proteinase K 20 min, RT	1:500	ImmPRESS anti-mouse 15 min
			45 min	DAB, hematoxylin counterstain
CD163 (ED2)	Thermo Fisher Scientific	Proteinase K 20 min, RT	1:100	ImmPRESS anti-mouse 10 min
			60 min	DAB, hematoxylin counterstain
IBA1 rabbit polyclonal	Wako Pure Chemical Corp.	0.01 M citrate buffer, pH 6.0	1:500	EnVision anti-rabbit 40 min
		110°C 5 min	30 min	DAB, hematoxylin counterstain
MRC1 rabbit polyclonal	Abcam	1 mM EDTA, 0.1% Tween 20, pH 8	1:1000	EnVision anti-rabbit 40 min
		16 h 60°C	60 min	Vector NovaRED, hematoxylin counterstain
SIGLEC1 (ED3)	Bio-Rad	0.01 M citrate buffer, pH 6	1:200	ImmPRESS anti-mouse 30 min
		110°C 20 min	2 h	Vector VIP, no counterstain.

DAB, 3,3-diaminobenzidine.

**FIGURE 2.**
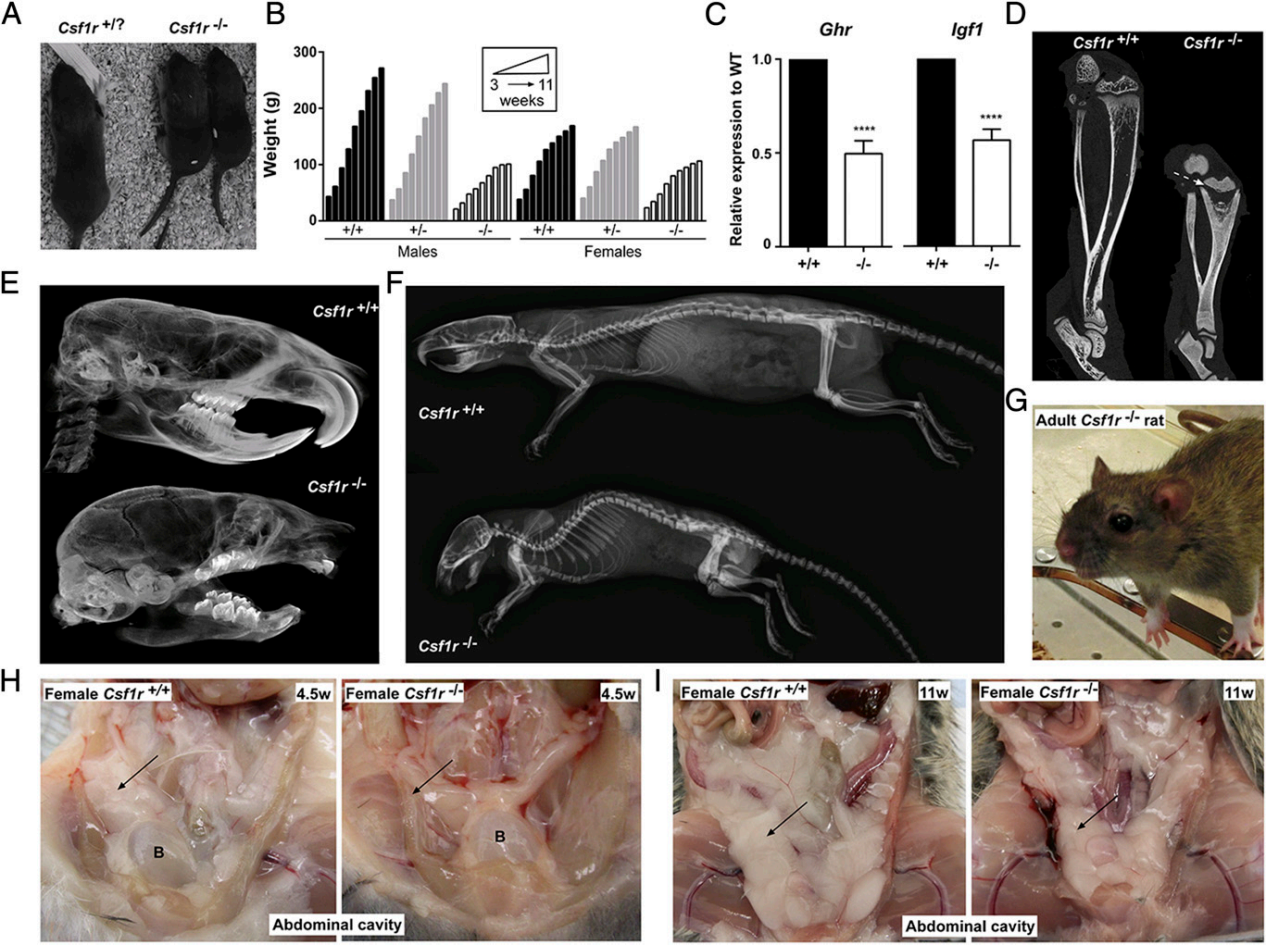
Gross phenotype of *Csf1r^−/−^* rats. (**A**) Two *Csf1r*^−/−^ rat pups and a littermate control at postnatal day 11. (**B**) Rats were weighed weekly following weaning at P21. For males, *n* = 5^+/+^, 8^+/−^, and 3^−/−^. For females, *n* = 7^+/+^, 4^+/−^, and 8^−/−^. Animals culled prior to 11 wk of age were included in the analysis. Graph shows the mean + SEM. (**C**) Total RNA was isolated from livers of wild type (^+/+^) and *Csf1r*^−/−^ rats (*n* = 6) for analysis of *Ghr* and total *Igf1* expression via qRT-PCR. There were equal numbers of males and females, four animals were adults, and two were 18 d old in each group. Graph shows the mean + SEM for pairwise comparison with age- and sex-matched littermate controls. Absolute values did not differ markedly between males and females or ages. Significance compared with wild type is indicated by ****p < 0.0001 using a *t* test. (**D**) Lower limbs from 7-wk males were fixed in 10% buffered formalin and analyzed for bone density by μCT. Dotted arrow indicates growth plate. (**E**) Skulls from 7-wk males were scanned by μCT. (**F**) Rats were analyzed by radiography at 6 mo of age. μCT images are from females, which are representative of both sexes. (**G**) An adult female *Csf1r*^−/−^ rat with bulging eyes. Representative photograph of the abdominal cavities of 4.5-wk-old (**H**) and 11-wk-old (**I**) females. Arrow points to visceral fat in the wild type (left) and absence or reduction of visceral fat in *Csf1r*^−/−^ rat (right). Images are also representative of males. B, bladder.

*Csf1r^−/−^* rats were indistinguishable from their littermates at birth but were identified from P9–11 by the absence of tooth eruption, smaller body size, and shorter snout (Fig. 2A), similar to the reported phenotype of the *Csf1* mutant (*tl/tl*) rat (24). The frequencies of wild type, heterozygous, and homozygous genotypes at birth were 27, 52, and 21% (*n* = 266), respectively, not significantly different from the expected 1:2:1 ratio expected under Mendelian inheritance (*p* = 0.3). All female *Csf1r*^−/−^ rats survived beyond weaning (*n* = 22), but 23% of male *Csf1r*^−/−^ rats were lost postnatally (*n* = 8) without obvious clinical signs. *Csf1r*^−/−^ rats weighed less than their littermates at weaning, and their body weight plateaued after 10 wk. As in *Csf1r*^−/−^ mice, the differential increase in growth/body size normally seen in wild type males was abolished in the *Csf1r*^−/−^ mutant animals, most likely reflecting a lack of testosterone production (9) (Fig. 2B). *Tl/tl* rats lack circulating insulin-like growth factor 1 (IGF1; responsible for body growth) in the postnatal period, implying a link between CSF1R and growth hormone receptor (GHR) signaling (25). Mouse macrophages grown in CSF1 express high levels of *Igf1* mRNA and are likely to be the major extrahepatic source (25). We have confirmed this finding in rats (see below). In the *Csf1r*^−/−^ rats, the mutation also impacted upon the major source of *Igf1* in the postpubertal growth surge. Both *Ghr* and total *Igf1* mRNA levels were consistently reduced by around 50% in the liver of mutant rats compared with age- and sex-matched litter mate controls (Fig. 2C).

*Csf1r*^−/−^ rats were osteopetrotic, with reduced bone marrow (BM) cavities and disrupted growth plates in the tibia (Fig. 2D). Although *Csf1r*^−/−^ rats lack any visibly erupted teeth, μCT analysis of the skull clearly showed molars and rudimentary incisors as well as a domed skull (Fig. 2E). By 6 mo of age, a severe curvature of the spine was apparent (Fig. 2F). Ageing *Csf1r*^−/−^ rats developed further phenotypes, including bulging eyes (Fig. 2G), that secreted porphyrin. The secretions were likely a consequence of the inability to blink properly, causing corneal dryness and increased porphyrin production. Postmortem analysis revealed that both male and female *Csf1r*^−/−^ rats had an almost complete absence of visceral white adipose tissue at 4.5 wk (Fig. 2H) yet still developed interscapular brown adipose tissue (Supplemental Fig. 2A). White adipose tissue was detected in older *Csf1r*^−/−^ rats but was still greatly reduced when compared with littermate controls (Fig. 2I). As the rats aged, they developed increased respiratory rates, most likely a result of cranial bone defects limiting the space available for soft tissues such as the soft palate and tongue, similar to brachycephalic syndrome in certain dog breeds (26). Indeed, the tongues of *Csf1r*^−/−^ rats were the same size as littermate controls, despite their much smaller body size (Supplemental Fig. 2B). A subset of males exhibited a protruding penis, without any adverse effects on behavior or urination, with osteopetrosis of the penile bone being the most probable cause.

Aside from the obvious difference in viability between mutant rats and mice, many pleiotropic impacts of macrophage deficiency described in *Csf1* and *Csf1r* mutant mice (9, 27, 28) were not observed in the *Csf1r*^−/−^ rats. For example, they showed no evidence of sensory defects (sight, hearing, and smell were apparently normal), produced insulin in histologically normal pancreatic islets (Supplemental Fig. 2C), and retained Paneth cells in the intestines, which also showed normal villous architecture (Supplemental Fig. 2D).

### Analysis of blood from Csf1r^−/−^ rats

**FIGURE 3.**
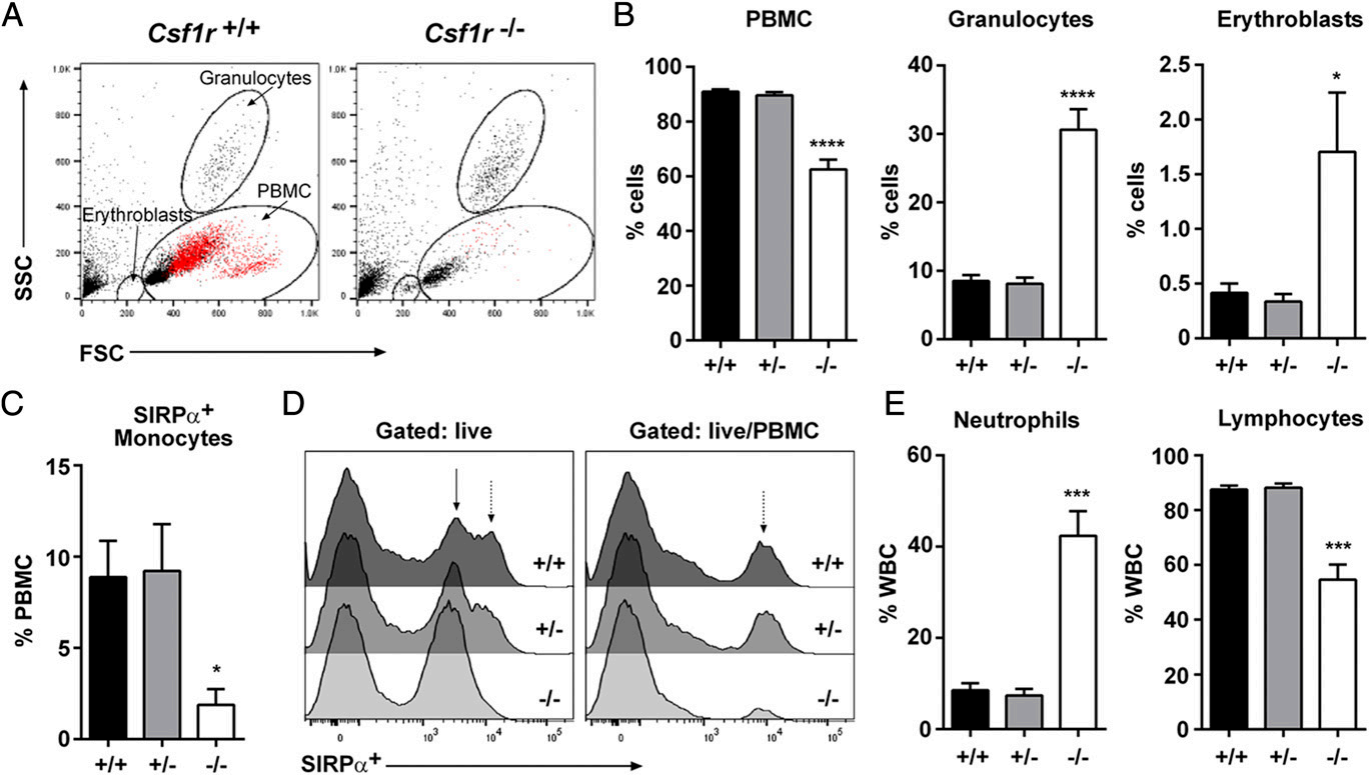
Analysis of whole blood. (**A**) Whole EDTA blood from adult rats was used to analyze forward versus side scatter (FSC/SSC) profiles by flow cytometry. SIRPα^+^ monocytes are colored red. (**B**) The percentages of PBMCs, granulocytes, and erythroblasts were determined by FSC/SSC profiles (*n* = 5 per genotype). (**C**) Cells were gated on SIRPα^+^ PBMC to determine the percentage of monocytes (*n* = 5 per genotype). (**D**) SIRPα expression was analyzed in whole EDTA blood gating on total live cells and live PBMC. Solid arrow highlights SIRPα^low^ granulocytes. Dotted arrow highlights SIRPα^high^ monocytes (*n* = 5 per genotype). (**E**) Whole EDTA blood was analyzed on an automated counter to determine the percentage of neutrophils and lymphocytes in WBCs (*n* = 6^+/+^, 10^+/−^, and 8^−/−^). Graphs show the mean + SEM. Significance compared with wild type is indicated by *p = 0.046 (erythroblasts) and 0.015 (monocytes), ***p < 0.0003, and ****p < 0.0001 using a *t* test.

**FIGURE 4.**
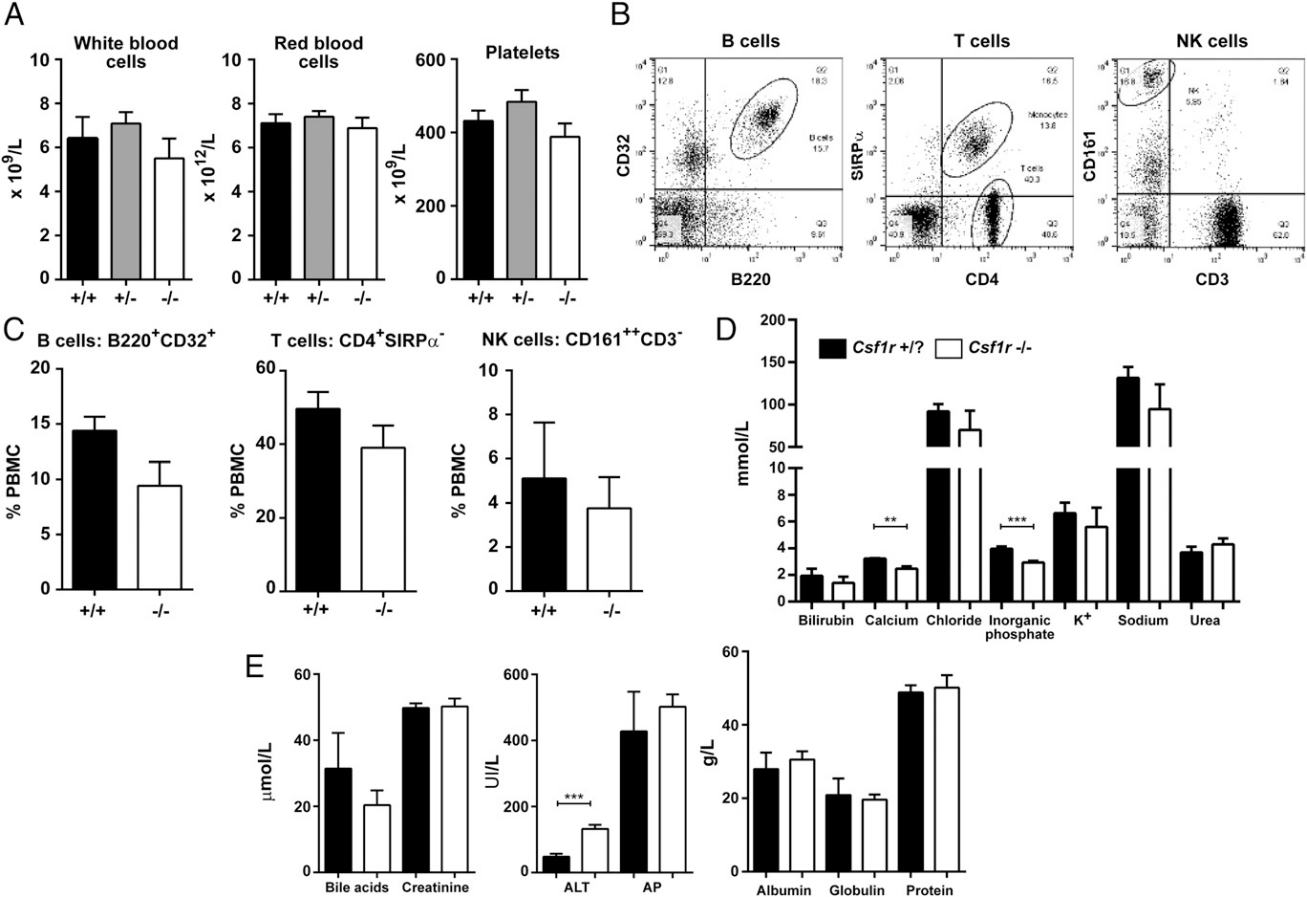
Further analysis of *Csf1r*-deficient rat blood. (**A**) Whole EDTA blood was used to determine the total number of WBCs, RBCs, and platelets from male and female rats aged between 6 and 12 wk (*n* = 6^+/+^, 11^+/−^, and 8^−/−^). (**B**) Whole EDTA blood was analyzed by flow cytometry to identify B, T, and NK cells. Dot plots show gating strategy for each cell type using *Csf1r* ^+/+^ blood. Cells were gated on the PBMC population by forward versus side scatter (FSC/SSC). Quadrants were determined with isotype controls (*n* = 5^+/+^ and 6^−/−^). (**C**) Graphs shows the mean + SEM. *p* = 0.09, 0.23, and 0.63 for B, T, and NK cells, respectively. (**D** and **E**) Serum from male and female rats aged 2–4 wk was analyzed (*n* = 5 per genotype). Graphs shows the mean + SEM. **p = 0.0018, ***p = 0.0010 (inorganic phosphate) and 0.0007 (ALT). ALT, alanine aminotransferase; AP, alkaline phosphatase, K^+^, potassium.

*Csf1r*^−/−^ rats had a significant decrease in PBMCs, whereas the percentages of granulocytes and erythroblasts were increased (Fig. 3A, 3B). By contrast with the CSF1 ligand–deficient *Csf1*^tl/tl^ rat (29), there was no evidence of thrombocytopenia, nor any change in total RBC or WBC count (Fig.4A). SIRPα^+^ monocytes were unaffected in the heterozygote but were almost undetectable in the homozygous mutant (Fig. 3C, 3D). Conversely, neutrophils were increased and lymphocytes were decreased (Fig. 3E) as reported previously in the *Csf1r*^−/−^ mice (9). There was no change in relative abundance of B, T, or NK cells within the lymphoid compartment (Fig. 4B, 4C). Blood albumin and electrolyte levels were unaffected by the mutation (Fig. 4D, 4E), with the exception of calcium and inorganic phosphate, which were both significantly reduced, most likely associated with deficient bone resorption.

### Fertility of Csf1r^−/−^ rats

**FIGURE 5.**
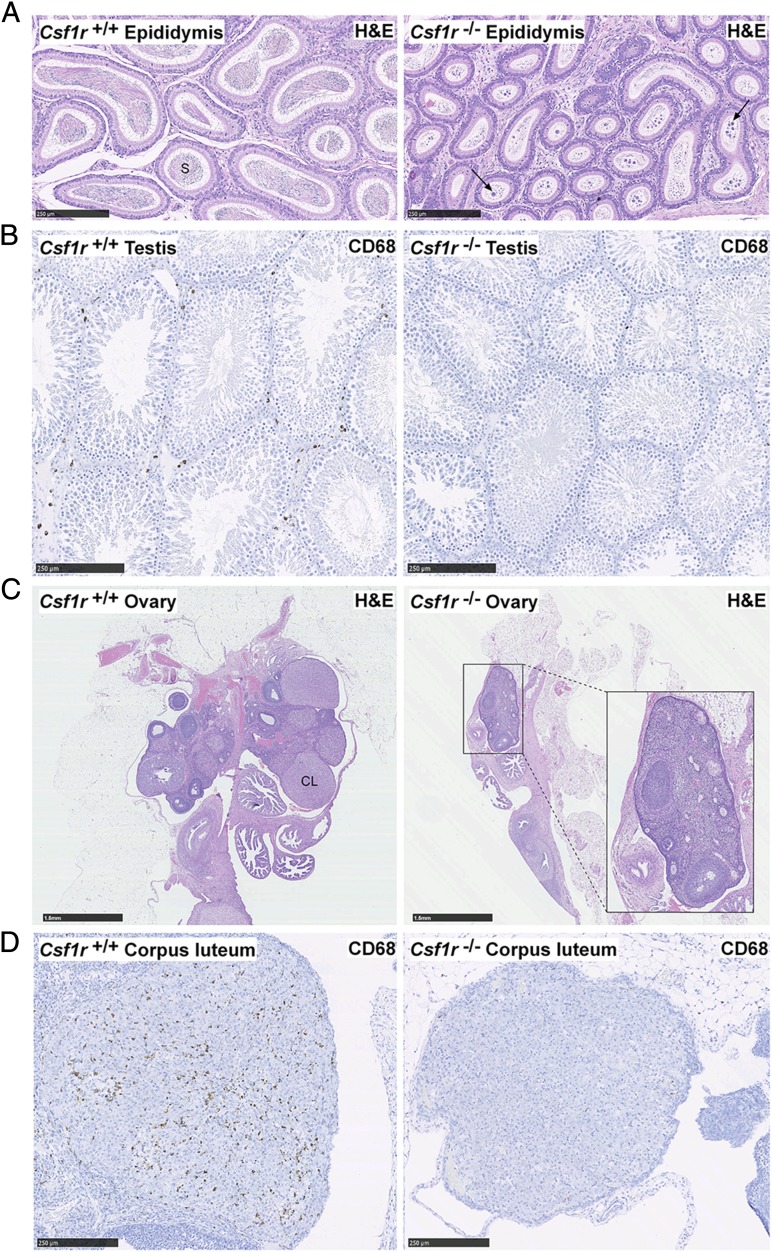
Histology of reproductive organs. Formalin-fixed and paraffin-embedded epididymides, testes, and ovaries were stained with H&E or an Ab against CD68. (**A**) Longitudinal sections of epididymides and (**B**) testes. Images are representative of six rats aged between 7 and 11 wk. Arrows point to atypical residual bodies. Scale bar, 250 μm. (**C** and **D**) Longitudinal sections of ovaries stained with H&E and CD68. Images are representative of seven rats aged 11–12 wk. Scale bar, 1.5 mm (C) and 250 μm (D). In wild type and *Csf1r*^+/−^ rats, an average of 5.8 (range 3–11) corpora lutea were observed per ovary in H&E-stained sections (*n* = 10) compared with only 0.7 (range 0–3) in *Csf1r*^−/−^ rats (*n* = 7). Whole slide images were produced with a NanoZoomer slide scanner and images were exported with NDP.view2 software (Hamamatsu Photonics). CL, corpus luteum, S, sperm.

*Csf1*^tl/tl^ male rats reach sexual maturity and were reported to be fertile (17). To date, male *Csf1r*^−/−^ rats have produced no pregnancies when placed with wild type females. Histological analysis revealed a severe reduction in the number of mature sperm cells in the lumen of the epididymis and a large number of atypical residual bodies, suggesting impaired maturation of spermatids (30) (Fig. 5A). The mAb ED1 recognizes rat CD68 and is commonly used to identify rat macrophages (31). In the testis of *Csf1r*^−/−^ males, there was a decrease in CD68^+^ interstitial macrophages (Fig. 5B), similar to that shown in mice treated with anti-CSF1R (32). The size difference between *Csf1r*^−/−^ females and adult male wild type rats precluded test mating on welfare grounds, but the females are also likely to be infertile. Their ovaries were greatly reduced in size, and corpora lutea were either absent or greatly reduced (Fig. 5C). The few corpora lutea that were observed lacked CD68^+^ macrophages (Fig. 5D), which are known to regulate corpus luteum development in mice (33).

### Analysis of tissue macrophages in Csf1r^−/−^ rats

**FIGURE 6.**
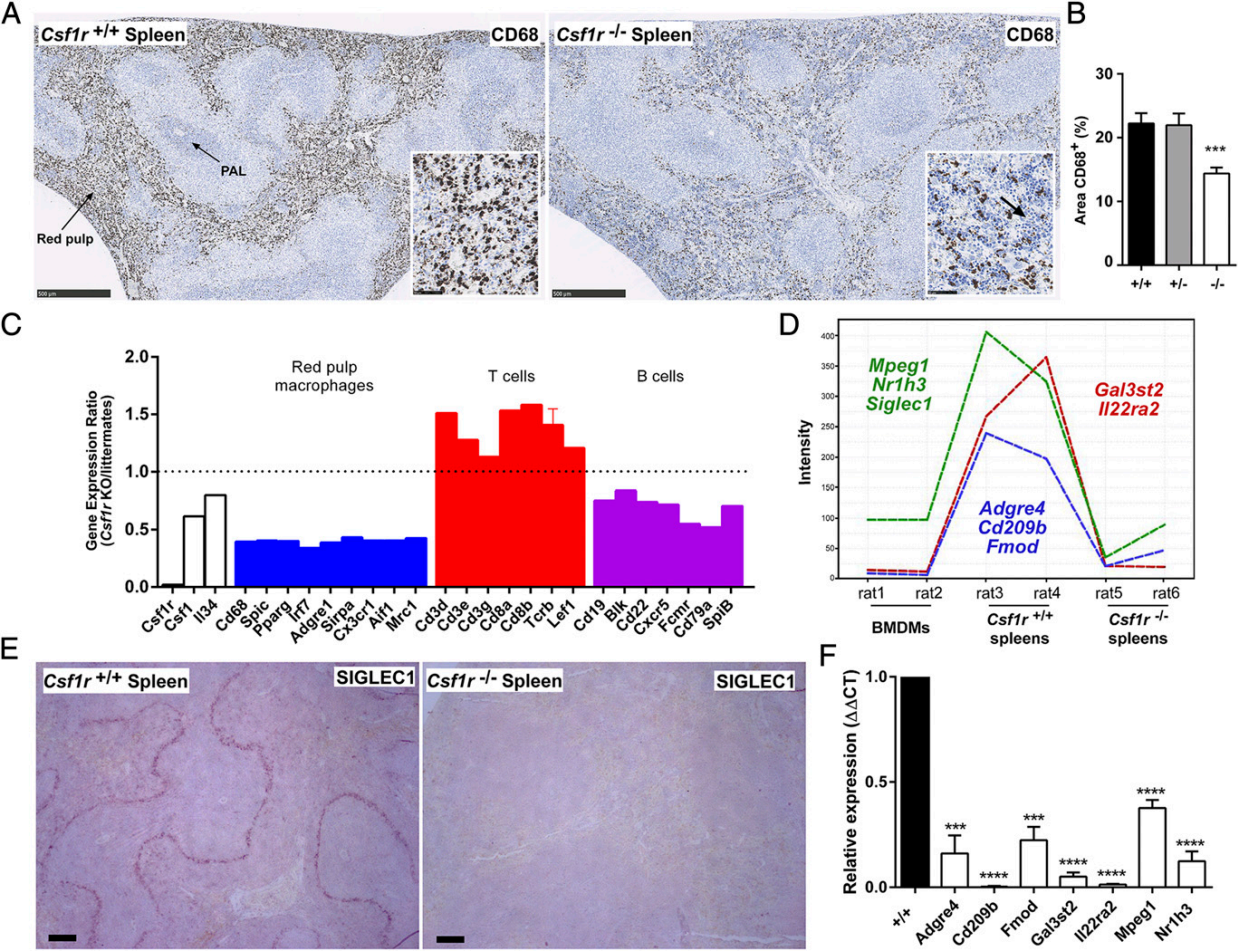
Analysis of spleens in *Csf1r*^−/−^ rats. (**A**) Formalin-fixed and paraffin-embedded spleens were stained with an Ab against CD68. Scale bar, 500 μm. Inset shows an area of red pulp. Scale bar, 50 μm. Arrow points to infiltration of lymphocytes in *Csf1r*^−/−^ inset. Whole slide images were produced with a NanoZoomer slide scanner and jpeg files exported with NDP.view2 software. PAL, periarteriolar lymphoid sheath. (**B**) The area of CD68^+^ cells in the red pulp was determined from 10 images per spleen (at original magnification ×80) using ImageJ (Fiji) (*n* = 10^+/+^, 8^+/−^, and 13^−/−^). Graph shows mean + SEM. Significance compared with wild type is indicated by ***p = 0.0002 using a *t* test. (**C**) Microarray data from spleens were used to determine expression ratios for genes associated with red pulp macrophages and T and B cells. Graph shows mean + SEM for genes with multiple probes. (**D**) Gene expression plot generated in Graphia Pro highlighting splenic macrophage-specific genes which were downregulated in *Csf1r*^−/−^ rats. (**E**) Formalin-fixed and paraffin-embedded adult spleens were stained with an Ab against SIGLEC1. Scale bar, 100 μm. Images are representative of 2^+/+^ and 3^−/−^ rats and two repeat experiments. (**F**) Total RNA was isolated from spleens of adult wild type (^+/+^) and *Csf1r*^−/−^ rats (*n* = 3) for analysis of candidate marginal zone macrophage-associated gene expression identified in (D) via quantitative PCR. Graph shows mean + SEM. Significance compared with wild type is indicated by ***p = 0.003 and ****p < 0.0001 using a *t* test.

There was a reduction of CD68^+^ macrophages in both the red pulp and the periarteriolar lymphoid sheath in spleen compared with wild type and heterozygous rats (Fig. 6A, 6B). This decrease was partly due to extensive infiltration of the red pulp by small mononuclear cells. There were no nucleated RBCs and no obvious increase in the number of megakaryocytes (Fig. 6A). Hence, there was no evidence of the extramedullary hematopoiesis that occurs in *Csf1r* mutant mice in the absence of a BM cavity (9). To evaluate the impact of the mutation on spleen cell populations, we compared gene expression profiles from *Csf1r*^+/+^ and *Csf1r*^−/−^ rats with BM-derived macrophages (BMDM). The full data are provided in Supplemental Table I. The set of 543 transcripts with expression ratios from 0.02 (*Csf1r*) to 0.5 (calculated as the ratio of expression in *Csf1r*^−/−^ to expression in littermates) (Supplemental Table I) includes CD68 (consistent with Fig. 6A), transcription factors (*SpiC*, *Pparg*), and the majority of markers of splenic red pulp macrophages including *Adgre1* (F4/80) (Fig. 6C). The reduction in the relative abundance of these transcripts (and by inference, the red pulp macrophages) may be partly attributable to the overall expansion of the lymphoid populations. The increased relative expression of T cell–associated genes (Fig. 6C) indicates that this expansion is biased toward T lymphocytes, whereas B cell markers were all reduced (Fig. 6C). A similar bias toward T cells was reported when *Csf1* was disrupted in mice (34). The spleen contains multiple different mononuclear phagocyte populations occupying specific niches, including the red pulp macrophages, marginal zone macrophages and metallophils, tingible body macrophages, and classical dendritic cells (35). The network analysis tool Graphia Pro (formerly BioLayout *Express*^3D^) was used to identify spleen-specific, macrophage-related genes that were downregulated with the loss of *Csf1r*. The *Csf1r*-dependent genes identified included markers of the separate marginal zone (*Siglec1*) and marginal zone metallophilic (*CD209b*) macrophages (Fig. 6D). Complete loss of the former population was confirmed via immunohistochemistry with an anti-SIGLEC1 Ab (Fig. 6E). The selective loss of CD209b, as well as a number of other candidate marginal zone-associated transcripts discussed below, was confirmed by qRT-PCR (Fig. 6F). The selective loss of the marginal zone macrophages may explain the disrupted splenic architecture and the migration of lymphocytes into the red pulp.

**FIGURE 7.**
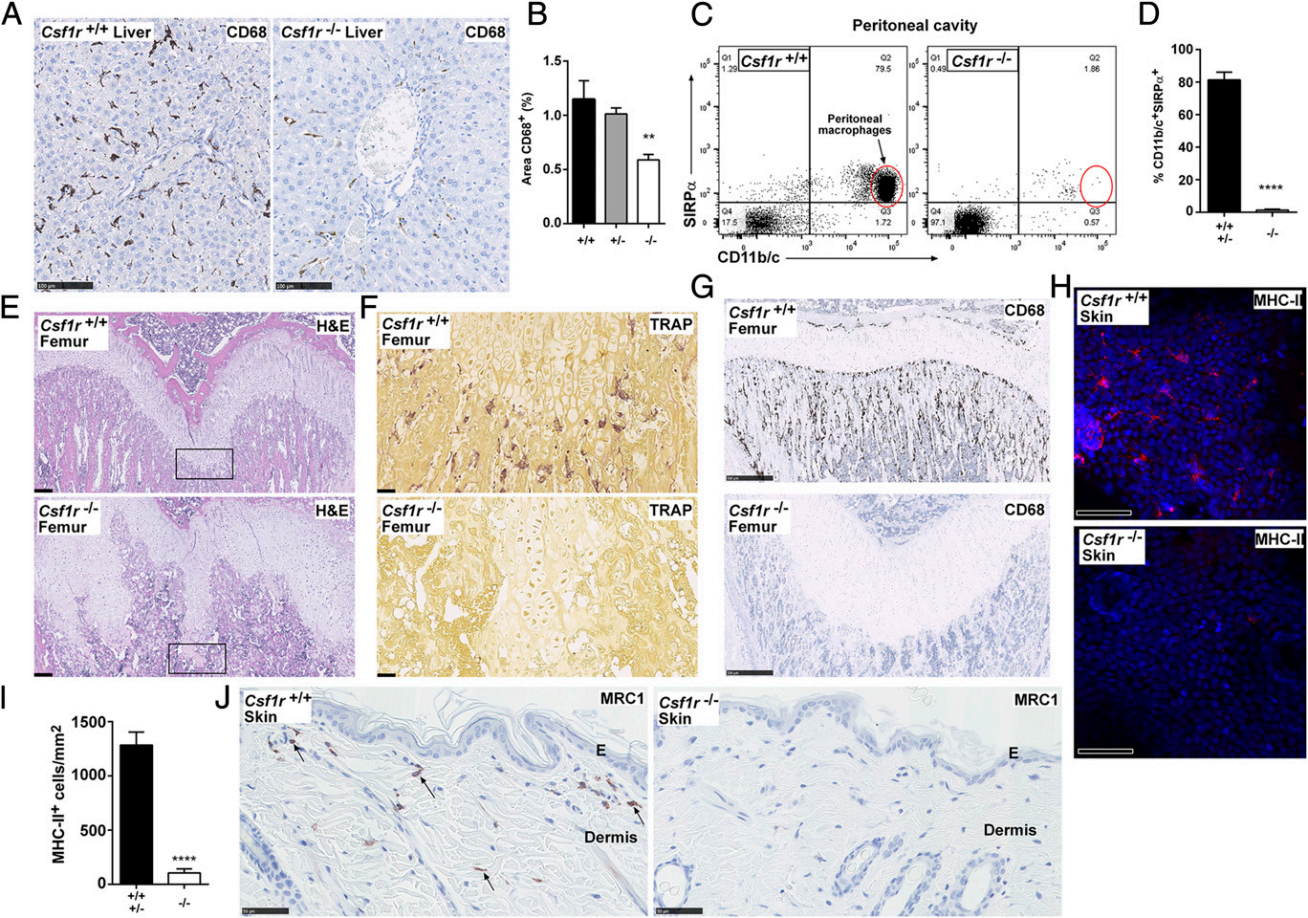
Analysis of tissue macrophages in *Csf1r*-deficient rats. (**A**) Formalin-fixed and paraffin-embedded livers were stained with an Ab against CD68. Whole slide images were produced with a NanoZoomer slide scanner and exported as jpeg files with NDP.view2 software. Scale bar, 100 μm. (**B**) The area of CD68^+^ cells was determined from 10 images per liver (at original magnification ×20) using ImageJ (*n* = 7^+/+^, 4^+/−^, and 9^−/−^). Graph shows mean + SEM. Significance compared with wild type is indicated by **p = 0.0035 using a *t* test. (**C**) Peritoneal cavity cells were analyzed by flow cytometry for expression of SIRPα and CD11b/c. Dead cells were excluded with propidium iodide, and quadrants were set with isotype controls. (**D**) Graph shows mean + SEM of CD11b/c^+^SIRPα^+^ peritoneal macrophages. Significance compared with wild type/heterozygotes is indicated by ****p < 0.0001 using a *t* test (*n* = 3). Decalcified femurs from adult rats were either stained with (**E**) H&E or stained for expression of (**F**) TRAP and (**G**) CD68. The boxed growth plate in (E) shows the area of TRAP staining. Images are representative of five rats per genotype. Scale bar, 200 μm (H&E), 500 μm (CD68), or 50 μm (TRAP). Whole slide images were produced with a NanoZoomer slide scanner and images exported with NDP.view2 software. (**H**) Epidermal sheets were prepared from the ears of adult rats and immunostained for MHC-II (red). Nuclear staining was performed using Hoechst 33258 (blue). Epidermal sheets were imaged as z-stacks (25 μm) using a Zeiss LSM710 confocal microscope. MHC-II and nuclei signals were acquired with 633 and 405 nm lasers, respectively. (**I**) Maximum intensity projections were produced from the z-stacks and MHC-II^+^ cells were quantified as per (102). Graph shows mean +SEM. Significance compared with wild type/heterozygotes is indicated by ****p < 0.0001 using a *t* test. Images are representative of four rats per genotype, two z-stacks analyzed per rat. Scale bar, 50 μm. (**J**) Formalin-fixed and paraffin-embedded dorsal skin was immunostained for MRC1 expression. Image is representative of 2^+/+^ and 3^−/−^ adult rats per genotype and two repeat experiments. Arrows point to MRC1^+^ dermal macrophages. Whole slide images were produced with a NanoZoomer slide scanner and images exported with NDP.view2 software. Scale bar, 50 μm. E, epidermis.

In the liver, CD68^+^ Kupffer cells were unaffected in the heterozygotes but partially depleted in *Csf1r*^−/−^ rats. The residual CD68^+^ cells appeared smaller and less ramified, but there was otherwise no gross change in liver size (relative to body weight) or architecture (Fig. 7A, 7B). Liver enzymes, alanine aminotransferase, and alkaline phosphatase were marginally increased in the circulation, but bile acids and bilirubin were unchanged and there was no histological evidence of injury (Fig. 4D, 4E). As in the *Csf1r*^−/−^ mice (9), peritoneal macrophages were absent in the *Csf1r*^−/−^ rats (Fig. 7C).

To examine effects on bone architecture, femurs were stained for TRAP (also known as ACP5) and CD68. As noted also in Fig. 2D, despite the cortical thickening and increased trabecular bone, the *Csf1r*^−/−^ rats retained a substantial BM compartment. This likely explains the lack of extramedullary hematopoiesis in the spleen. Osteoclasts were entirely deficient in *Csf1r*^−/−^ rats (Fig. 7F), as reported in *Csf1*^tl/tl^ rats (17), whereas in *Csf1r*^−/−^ mice TRAP^+^ cells are retained, albeit at a lower frequency (9). The surface of bone in mice and humans contains a population of specialized osteal macrophages, intercalated among the osteoblasts (36). Abundant osteal macrophages were detected with CD68 staining in rat bone, and they were completely absent in bones from the *Csf1r*^−/−^ animals (Fig. 7G).

In mice, IL-34 is also expressed in the epidermis and is required for the development of the MHC-II–positive epidermal Langerhans cells (37). In the *Csf1r*^−/−^ rats. MHC-II^+^ Langerhans cells, visualized in epidermal sheets, were almost completely depleted (Fig. 7H, 7I); the few remaining cells were only weakly MHC-II^+^. Dermal macrophages were also absent in *Csf1r*^−/−^ rats as detected by CD206 (mannose receptor, MRC1) expression (Fig. 7J).

### Analysis of Csf1r^−/−^ rat brains

**FIGURE 8.**
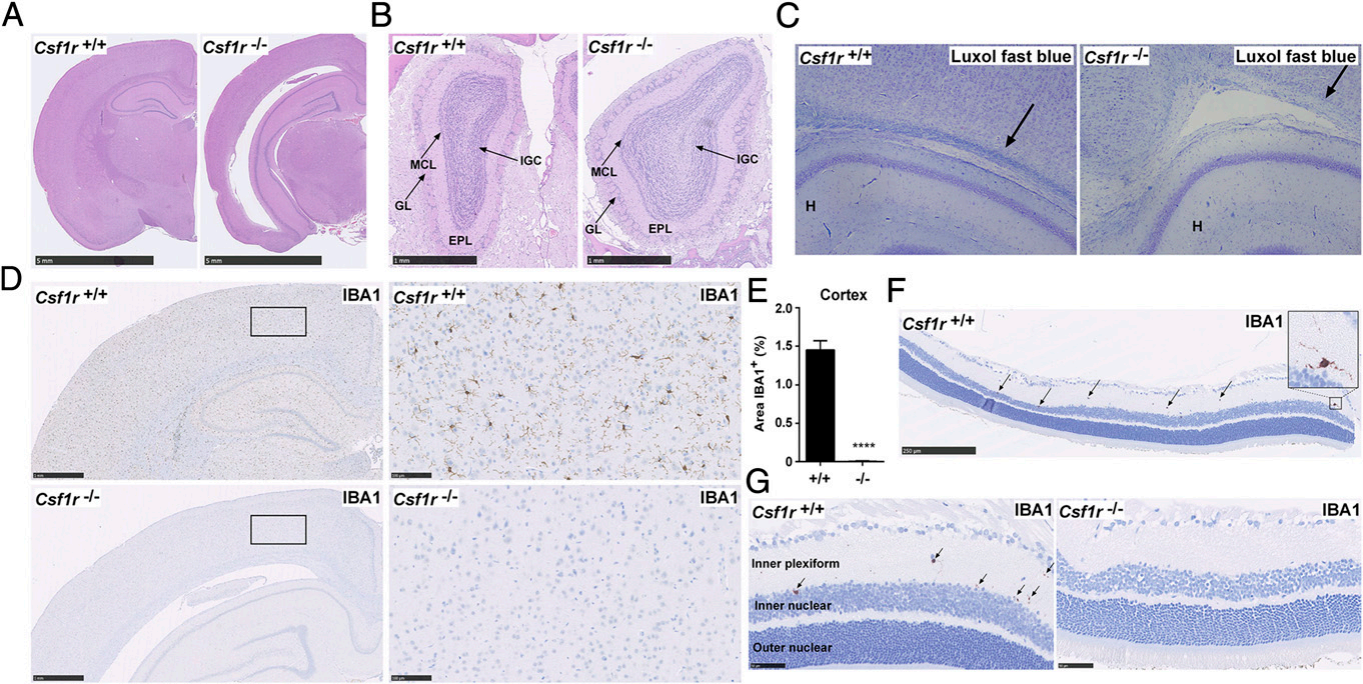
Analysis of brains from *Csf1r*^−/−^ rats. All brains were formalin fixed and paraffin embedded for histology and immunohistochemistry. (**A**) Brains from 11-wk-old rats were stained with H&E. Images are representative of seven rats per genotype. Scale bar, 5 mm. (**B**) Adult heads were fixed in formalin and stained with H&E following EDTA decalcification. Image shows olfactory bulbs in situ. Scale bar, 1 mm. Image representative of seven rats per genotype. EPL, external plexiform layer; GL, glomerular layer; IGC, internal granular cell layer of the olfactory bulbs; MCL, mitral cell layer. (**C**) Formalin-fixed brains from 3-wk-old rats were stained with Luxol fast blue. Arrows point to myelin preservation and myelin pallor in wild type and mutant rats, respectively. Image of three rats per genotype. (**D**) Adult brains (8–14 wk) were stained with an Ab against IBA1. Scale bar, 1 mm (left) or 100 μm (right). (**E**) For each rat, 10 20× images of the cortex were analyzed for the percentage of IBA1^+^ staining using ImageJ (*n* = 7 per genotype). Graphs show mean + SEM. Significance compared with wild type is indicated by ****p < 0.0001 using a *t* test. (**F** and **G**) Formalin-fixed eyes were stained with an Ab against IBA1. Arrows point to IBA1^+^ microglia in the inner plexiform layer of the retina. Images are representative of two rats per genotype, two repeat experiments. Scale bar, 250 μm (F) and 50 μm (G).

**FIGURE 9.**
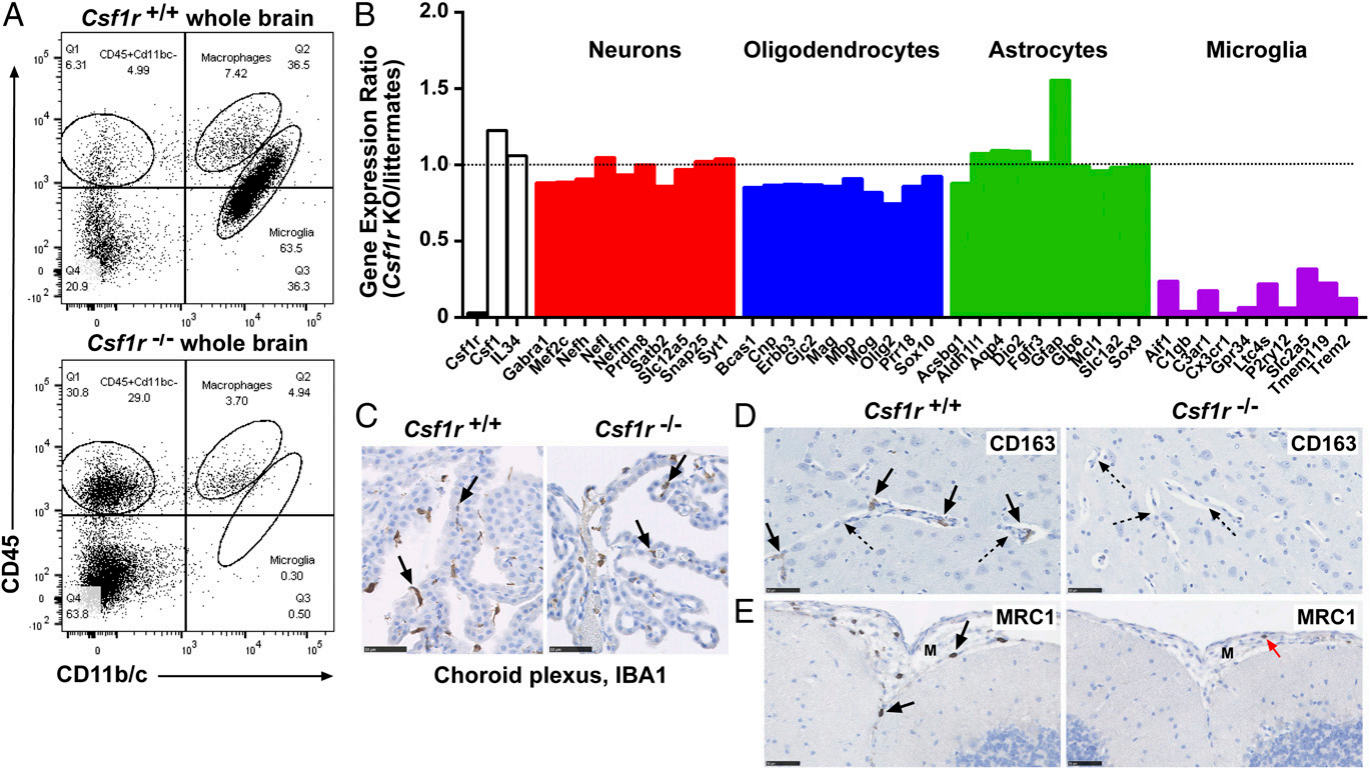
Further analysis of brains from *Csf1r*^−/−^ rats. (**A**) Single-cell suspensions of brain were depleted of myelin and analyzed by flow cytometry for CD11b/c and CD45 expression. Dead cells were excluded with propidium iodide, and blood granulocytes were excluded from *Csf1r*^−/−^ samples via forward versus side scatter (FSC/SSC). Quadrants were determined using isotype controls. Image is representative of six rats per genotype. (**B**) Microarray data from dissected striatum, hippocampus, olfactory bulbs, and pituitary gland were used to determine expression ratios for genes associated with the major cell populations of the brain. (**C**) Choroid plexuses from adult brains stained with an Ab against IBA1. Images are representative of seven rats per genotype. Arrows point to IBA1^+^ choroid plexus macrophages. Sclae bar, 50 μm (**D**) Adult brains were stained with an Ab against CD163. Images are representative of seven rats per genotype. Solid arrows point to CD163^+^ perivascular macrophages. Dotted arrows point to blood vessels. Scale bar, 50 μm. (**E**) Adult brains were stained with an Ab against MRC1. Images are representative of seven rats per genotype. Solid arrows point to MRC1^+^ meningeal macrophages in the *Csf1r*^+/+^ rat brain. Red arrow points to a monocyte in the *Csf1r*^−/−^ meninges (M). Scale bar, 50 μm. Whole slide images were produced with a NanoZoomer slide scanner and analyzed with NDP.view2 software (Hamamatsu Photonics).

The brains of *Csf1r*^−/−^ rats at 11 wk of age revealed few abnormalities apart from some enlargement of the lateral ventricles (Fig. 8A). Whereas *Csf1r*^−/−^ mice had hollow and fragile olfactory bulbs (27), these were intact and apparently normal in the rat (Fig. 8B). Minor loss of myelin was apparent at the level of the corpus callosum (Fig. 8C). *Csf1r*-deficient mice had a gross deficiency of microglia, detected with the microglial marker, IBA1 (27). IBA1^+^ cells with ramified morphology were similarly not detectable in any region of the brain (Fig. 8D) or in the plexiform layers of the retina (Fig. 8F, 8G) in adult *Csf1r*^−/−^ rats. To confirm that this deficit was due to a loss of microglia, rather than loss of the marker, cells extracted from total brain tissue were analyzed (21). CD11b/c^+^CD45^low^ microglia were entirely absent in *Csf1r*^−/−^ rats (Fig. 9A), and CD11b/c^+^CD45^hi^ macrophages (normally a minor component in brain digests) were decreased. The population of CD11b/c^−^CD45^hi^ cells was increased in *Csf1r*^−/−^ rats. We attribute this to blood contamination as a result of incomplete perfusion (discussed below).

The brain, like the spleen, contains several macrophage populations in addition to microglia (38). To further analyze the impact of *Csf1r* mutation on brain myeloid populations and on other brain cells, we profiled gene expression in the striatum, hippocampus, olfactory bulbs, and pituitary gland. The full dataset is provided in Supplemental Table I. There was no global change in expression of generic neuron-associated genes in *Csf1r*^−/−^ rats shared by the four brain regions examined, but there was some evidence of region-specific impacts, discussed below.

The set of 131 transcripts with expression ratios from 0.03 (*Ctss*, *Cx3cr1*, and *Csf1r*) to 0.5 (Supplemental Table I) contains many microglia-enriched transcripts identified in mice (Fig. 9B) (39–41) and rats (42). By contrast to the loss of microglia-specific markers, many macrophage-associated marker genes remained readily detectable in *Csf1r*^−/−^ brains (e.g., *Cd14* and *Fcgr1a* encoding CD64). We attempted to locate residual macrophages with known markers. As in the *Csf1r*-deficient mouse (27), there were no detectable CD68^+^ cells in *Csf1r*^−/−^ rat brains via immunohistochemistry (data not shown). *Csf1r*^−/−^ rats retained IBA1^+^ macrophages in the choroid plexus (Fig. 9C) but lacked detectable CD163^+^ perivascular macrophages (43) and MRC1^+^ meningeal macrophages (38) (Fig. 9D, 9E).

The blood–brain barrier was intact in *Csf1r*^−/−^ rats, as evident from Evans blue exclusion. Interestingly, there was an obvious reduction in Evans blue staining in the periphery of *Csf1r*^−/−^ rats, suggesting a subtle vascular phenotype. Evans blue staining in the spleen was also reduced (Fig. 9A), despite no differences in levels of the carrier albumin (Fig. 4E), potentially highlighting the known role of macrophages in angiogenesis (reviewed in Ref. 44).

### Network analysis of gene expression in the spleen of Csf1r-deficient rats

In previous studies of the effect of CSF1 treatment on macrophage numbers and gene expression profiles in the liver, BMDM were used as an external reference to identify genes that were enriched in macrophages relative to the tissue (45). These genes provided a signature of CSF1-dependent expansion of the macrophage population detectable in total mRNA from the liver (45). To provide a similar reference for the rat, BMDM were generated by cultivation in a medium containing recombinant human CSF1 for a period of 7 d as described for mouse (45), mRNA was isolated, and transcript expression was profiled in parallel with tissue mRNA from adult rats.

To identify the set of genes that were spleen enriched, based upon comparison with BMDM grown in CSF1, the wild type, heterozygous, and homozygous *Csf1r* mutant expression profiles were combined with BMDM and clustered using Graphia Pro. Surprisingly, one of the two heterozygous mutant animals had much lower expression of *Csf1r*, albeit higher than in the knockout. (Supplemental Table I).

**FIGURE 10.**
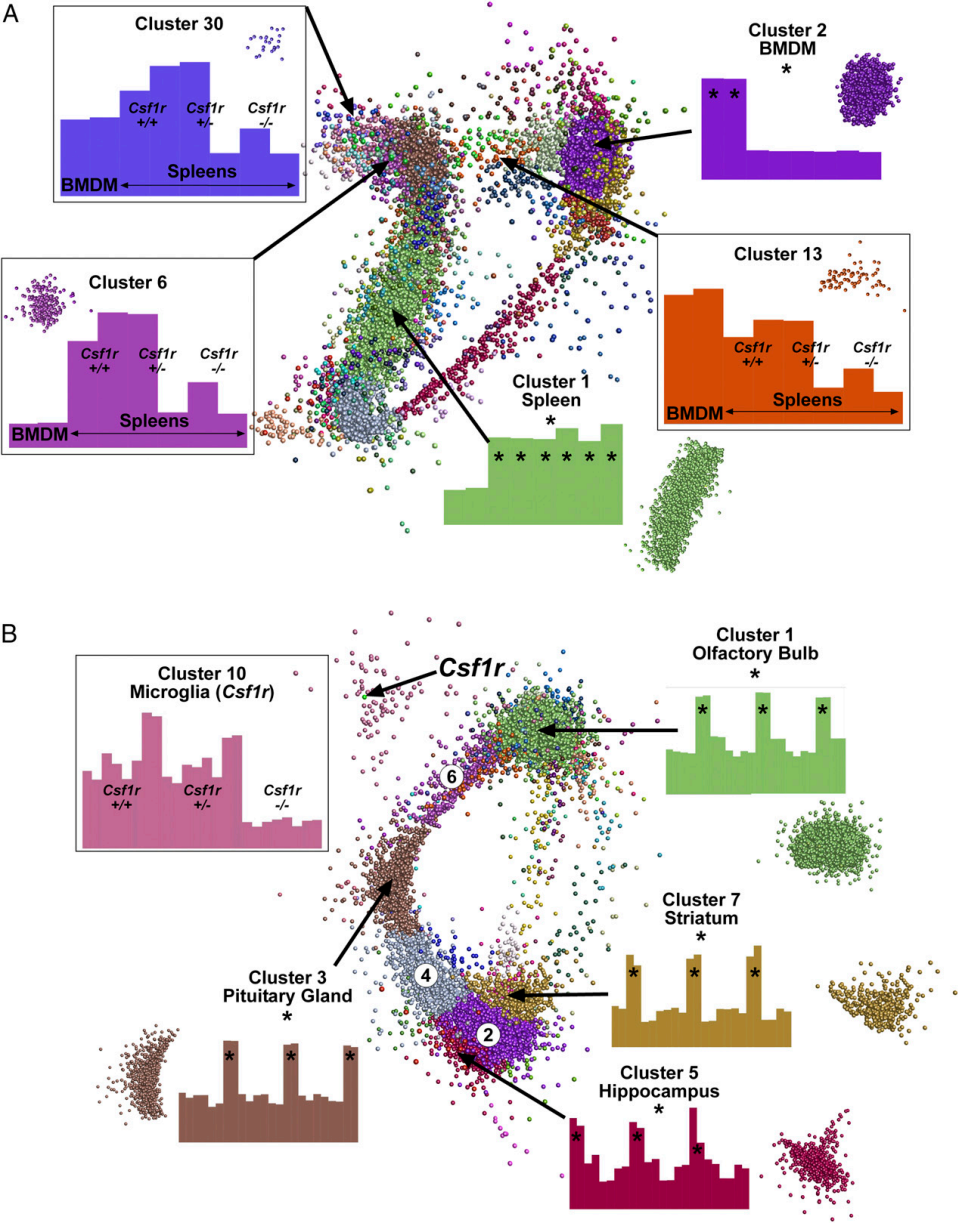
Network analysis of gene expression in the spleen and brains of *Csf1r* deficient rats. RMA-normalized microarray data from Supplemental Table I was analyzed with Graphia Pro. Edges have been removed for ease of visualization. Nodes allocated to the same cluster are the same color. Histograms show the averaged expression patterns of all genes in the cluster. Boxed clusters refer to genes affected by loss of *Csf1r*. Unboxed clusters refer to genes that are tissue specific (*). (**A**) Key clusters from spleen. All known genes in which no sample reached an intensity of 20 were excluded. Analysis was performed at a Pearson correlation coefficient ≥0.95 (12,305 nodes making 1,746,925 edges). Clustering was performed at an inflation of 2.0 with a minimum cluster size of 10. (**B**) Key clusters from brain. All known genes in which no sample reached an intensity of 20 were excluded. Analysis was performed at a Pearson correlation coefficient ≥0.85 (11,833 nodes making 3,617,804 edges). Clustering was performed at an inflation of 2.0 with a minimum cluster size of 10. Three clusters (circled numbers) shared gene expression with multiple brain regions: cluster 2 (striatum and hippocampus), cluster 4 (pituitary gland, striatum, and hippocampus), and cluster 6 (olfactory bulb and pituitary gland). Histograms for these clusters are shown in Supplemental Table I.

The network graph divided into two major aggregates, essentially comprising clusters of macrophage-enriched and spleen-enriched transcripts. Fig. 10A shows the clustered nodes for the main element in the layout graph.

Five clusters of specific interest are among those listed in Supplemental Table I, together with their average expression profile. The largest cluster, cluster 1 (2171 genes; green nodes), was strongly enriched in the spleen compared with BMDM and not sensitive to *Csf1r* mutation. The second largest cluster (cluster 2, 1986 genes; purple nodes) was strongly enriched in the BMDM and also was not affected by the loss of *Csf1r*. This list is a complex mixture of genes associated with phagocyte biology and include *Adgre1* (F4/80), *Itgam* (CD11b), components of the vacuolar ATPase (*Atp6v* family), *Fcgr*, and *Clec* families. The existence of this cluster indicates that there is a substantial residual macrophage population in the spleen of *Csf1r^−/−^* rats, and they share with BMDM the majority of the generic macrophage transcriptome. That conclusion is consistent with the residual CD68 staining in Fig. 6A and 6B. Also within cluster 2, there were more general anabolic/metabolism-associated genes involved in mitochondria (e.g., *Nduf*, *Cox*, *Mrpl* families) and RNA/protein synthesis. The enrichment of these genes in cluster 2 reflects the difference between a pure growing macrophage population and the splenic lymphoid population, in which most of the cells are relatively metabolically inactive.

*Csf1r*-dependent clusters varied in their level of spleen enrichment. The expression profile of cluster 6 (238 genes) was much higher in spleen than in BMDM and downregulated in the knockout and in the low *Csf1r*-expressing heterozygote. Cluster 30 (24 genes) had a similar expression profile to cluster 6, but with higher relative expression in BMDM. The spleen-specific cluster 6 contained the signature gene for the marginal zone macrophages (*Siglec1*) which was among the most downregulated with the loss of *Csf1r* (Supplemental Table I). The IL-22–binding protein, encoded by *Il22ra2*, has recently been studied in detail in Peyer patch, where it is highly expressed by myeloid cells underlying follicle-associated epithelium, regulates IL-22 signaling, and indirectly affects bacterial uptake (46). The loss of this transcript was confirmed by qRT-PCR (Fig. 6F).

The gene expression profile in the spleen of *Csf1r*-deficient rats is consistent with almost complete loss of the marginal zone macrophage populations. These cells, and the related Ag capture cells in the subcapsular sinus of lymph nodes, were also depleted in *op/op* mice (47). The data suggest that relatively few *Csf1r-*dependent genes are absolutely restricted to the marginal zone.

Genes that are less spleen specific but are nevertheless *Csf1r* dependent (e.g., those in cluster 13, including *Sirpa*) may be shared by macrophages of the marginal zone and red pulp, or specifically associated with red pulp macrophages. Overall, the main impact of *Csf1r*-deletion is to compromise spleen-specific macrophage differentiation.

### Network analysis of gene expression in the brain of Csf1r-deficient rats

As with the spleen, the first pass analysis of gene expression data for two brain regions was to determine the expression ratios (ratio of *Csf1r*^−/−^/littermates) for each of the transcripts on the arrays in each of the tissues. There was no global change in expression of generic neuron-associated genes in *Csf1r*^−/−^ rats shared by the brain regions examined, but there was some evidence of region-specific impacts (Supplemental Table I). There were several overlaps between the *Csf1r*-dependent genes in the brain regions and spleen, including *Gpr31*, *Gpr34*, *Cd33*, *Tmem119*, *Tmem176a*, *P2ry13*, *Pld4*, *Clec4a1*, and *C1q* genes, but most were not shared, consistent with the tissue-specific adaptation of *Csf1r*-dependent macrophages in the two locations. Fig. 10B shows the network graph for the combined analysis of the four brain regions; gene lists are provided in Supplemental Table I. The largest cluster was cluster 1 (4283 genes), containing olfactory bulb–associated genes. Other region-specific clusters were cluster 3 (pituitary gland; 878 genes), cluster 5 (hippocampus; 513 genes), and cluster 7 (striatum; 384 genes). The second largest cluster (cluster 2; 1253 genes) contained genes that were more highly expressed in both hippocampus and striatum. Two other main clusters also shared highly expressed genes between tissues: cluster 4 (pituitary gland, striatum and hippocampus; 757 genes) and cluster 6 (pituitary gland and olfactory bulb; 471 genes). None of these clusters showed any evidence of genotype association. Cluster 10 (107 genes) contained genes that were downregulated in the *Csf1r*^−/−^ rats in all brain regions, including *Csf1r* itself (Fig. 10B). This set contains most known microglia-specific transcripts and is presumably attributed to the loss of microglia.

## Discussion

The *Csf1r* knockout rats described in this article join a small set of rat knockout lines that have been produced via homologous recombination in ESC [(48) and reviewed in Ref. 13]. The comparison between rat and mouse mutant phenotypes supports the view that the rat, in many cases, provides a superior model for studying human genetic disease. For example, p53–deficient rats exhibited a more human-like tumor spectrum than mice (49), and dystrophin mutant rats displayed heart defects which were more similar to the human condition Duchenne muscular dystrophy (50, 51). The phenotype of *Csf1r*^−/−^ rats is clearly very different from mice, and because of the improved viability, we have assayed phenotypes that could not be accessed in mice. In any insertional mutagenesis, or deletion, there is always the possibility of *cis*-acting impacts on neighboring genes. The two genes neighboring *Csf1r*, namely *Hmgxb3* and *Pdgrb*, are both robustly expressed in all three tissues examined by gene expression profiling, and there was no significant change in their expression in response to insertional mutagenesis of *Csf1r.*

A common trait between *Csf1r*^−/−^ rats and mice is increased bone density (osteopetrosis) attributed to either a reduction (mice) or complete absence (rats) of bone-resorbing osteoclasts. In humans, genetic susceptibility to disordered bone turnover in Paget disease has been linked to variation at the *Csf1* locus (52). In *Csf1*^tl/tl^ rats, there is a severe loss of osteoclasts in addition to the loss of osteoblasts (53). Unlike *Csf1*^tl/tl^ rats (54), the *Csf1r*^−/−^ rats developed a BM cavity, albeit reduced, and showed no evidence of extramedullary hematopoiesis in the spleen. Nevertheless, only monocytes were greatly reduced in the blood. We speculate that the loss of macrophages in liver and spleen increases the half-life of red cells, platelets, and neutrophils, compensating for reduced production by the BM. The receptor mutants also do not display the early onset deafness seen in *Csf1*^tl/tl^ rats, attributed to auditory ossicle abnormalities (55). The loss of osteoclasts in *Csf1r*^−/−^ rats is probably balanced in part by deficient calcification by osteoblasts. Osteoblasts on the bone surface interact intimately with a population of osteal macrophages, which are essential for intramembranous and endochondral ossification (36, 56). In the *Csf1r^−/−^* rat, there were no CD68^+^ osteal macrophages detectable on the bone surfaces (Fig. 7F). The difference between ligand and receptor mutations presumably reflects the role of the second ligand, IL-34. Both CSF1 and IL-34 (57) are produced by osteoblasts and contribute to osteoclastogenesis. It appears likely that IL-34 is essential for rat osteal macrophage development. Conversely, there is a lack of sperm development and fertility in male *Csf1r*^−/−^ rats, whereas *Csf1*^tl/tl^ male animals are fertile. This suggests that the alternative ligand, IL-34, has a specific function in testis development in the rat. Consistent with that hypothesis, *Il34* mRNA is highly expressed in rat testis and downregulated by candidate male contraceptives (58).

Other phenotypes shared between *Csf1r*^−/−^ rats and mice include postnatal growth retardation, lack of tooth eruption, reduced circulating monocyte numbers, defects in fertility, reduction in liver and peritoneal macrophages, and loss of Langerhans cells. The most obvious difference between *Csf1r*^−/−^ rats and mice is the greatly increased viability, relative lack of a severe brain phenotype, and absence of effects on the sensory nervous system, insulin/pancreatic islet development, and gastrointestinal tract in the rats, as noted above. These species-specific differences, especially the latter two, probably contribute to the improved postweaning survival of the rat knockout compared with the mouse. The gut phenotype in mice has been attributed to *Csf1r* expression by Paneth cells, which appeared to be absent in mutant mice, and *Csf1* signaling is proposed to indirectly control the differentiation of intestinal epithelial cells (59, 60). Treatment of mice with anti-CSF1R Ab completely depleted intestinal macrophage populations and lysozyme expression in the intestinal crypts and was associated with an increase in goblet cells (32). More recently, we demonstrated that *Csf1r* mRNA is not expressed in mouse Paneth cells, but macrophages intimately associated with the crypt control the differentiation of these cells and *Lgr5*^+^ stem cells. Whereas lysozyme expression was *Csf1r* dependent, other Paneth cell markers, including defensins, were not. In addition to increased goblet cell formation, anti-CSF1R treatment produced a complete loss of Ag-sampling M cells (61). In the rat *Csf1r* knockout, there was no effect on the cellularity of the lamina propria, no loss of Paneth cells, no increase in goblet cells, and no discernible change in villus architecture (Supplemental Fig. 2). In mice, the large intestinal macrophage population apparently depends upon continuous replenishment from circulating monocytes (62). In the *Csf1r* mutant rat, monocytes were greatly reduced, yet there was no apparent impact on the intestine.

In contrast, the almost complete loss of visceral fat in *Csf1r*^−/−^ rats at an early age is a novel phenotype that has not previously been noted in mice. The *Csf1*^op/op^ mouse is not deficient in adipose tissue but does have a reduction of macrophages in this tissue (63). Adipose tissue growth is related to growth hormone and IGF1 production (64). Hence, the loss of adipose tissue is most likely linked to the reduced GHR and IGF1 we observed in the liver and the consequent loss of circulating IGF1, as noted previously in *Csf1*^tl/tl^ rats (45). A previous study also noted the loss of GHR expression in the bone of *Csf1*^tl/tl^ rats (65).

Injection of Evans blue stain highlighted a deficiency in development of the peripheral vasculature in *Csf1r*^−/−^ rats. Notably, the apparent leakiness in the spleen (Supplemental Fig. 3B) appears to be associated with the marginal zone and may be an indirect consequence of the loss of the marginal zone macrophages. The *tl/tl* CSF1 mutant rats display impaired capillary proliferation in the femur, which can be rescued with CSF1 treatment (66). However, the peripheral blood vessels have not been examined in these rats.

The gene expression analysis in Fig. 6C and 6D allowed us to identify spleen-specific genes that were downregulated with the loss of *Csf1r*. The large majority of genes enriched in BMDM relative to spleen were not affected. Transcripts associated with classical dendritic cell differentiation, such as *Itgax*, *Zbtb46*, *Ly75*, or *Clec9a*, were also unaffected, suggesting that these cells are not *Csf1r* dependent. Those genes that were both spleen enriched and *Csf1r* dependent provide a surrogate indicator of the loss of specific cell populations. The marginal zone macrophages of the mouse have resisted isolation and characterization (41). The commonly studied marker genes *Siglec1* (marginal zone macrophages) and *CD209b* (marginal metallophils) (35) were almost ablated in *Csf1r*^−/−^ rats, and coregulated genes are implicated, by association, in marginal zone macrophage differentiation and function (Supplemental Table I). Very few genes show the same level of *Csf1r* dependence. One example is the IL-22–binding protein, encoded by *Il22ra2*. This transcript is highly expressed by myeloid cells underlying mouse follicle-associated epithelium, regulates IL-22 signaling, and indirectly affects bacterial uptake (46).

The marginal zone macrophage population is also absent from *Csf1^op/op^* mice (47). Marginal zone macrophages in mice are distinguished from red pulp macrophages by expression of the widely used macrophage marker, F4/80, encoded by *Adgre1* (previously *Emr1*) (67). *Adgre1* is also highly expressed by rat macrophages (Supplemental Table I), and the relative loss of expression in the knockout spleen (∼70%) is consistent with expression in the red pulp macrophages. The related gene, *Adgre4* (previously *Emr4*), was strongly spleen enriched and highly *Csf1r* dependent (Fig. 6F). ADGRE4 was shown to mediate binding to B lymphocytes (68), supporting the hypothesis that it may be a novel functional marginal zone marker.

The set of candidate marginal zone–enriched transcripts includes the transcription factor *Nr1h3* (aka *Lxrα*) (Fig. 6F), which is essential for both marginal zone macrophage populations in mouse (69). Other transcription factors coregulated with *Lxrα* (Supplemental Table I, cluster 6) included *Nfe2*, *Nr1d1*, *Rara*, *Klf2*, and *Tcf21*.

One report on *Nr1d1* in macrophages confirms the enriched expression in mouse splenic macrophages and a circadian oscillation (70). Regulated expression of the retinoic acid receptor, *Rara*, has also been associated with myeloid differentiation (71), and treatment of mice with retinoids led to an expansion of the marginal zone in spleen (72). *Tcf21* is required during development for the formation of a spleen (73). This *Csf1r*-dependent cluster also contained multiple growth factors of the TGFB (*Tgfb2*, *Tgfb3*) and BMP (*Bmp2, 3, 4, 6*) families, which could contribute to the differentiated phenotype of splenic macrophages (74, 75). Expression of the transcription factors *Tfec* and *Tcf7l2* was downregulated with the loss of *Csf1r* (Supplemental Table I). *Tfec* is a member of the MITF transcription factor family and is known to be macrophage enriched and PU.1 dependent (76). Analysis of the *Tfec*^−/−^ mouse suggested a role in IL-4–inducible expression of genes, including *Csf3r* (77), which was also downregulated in the *Csf1r*^−/−^ spleen. Our finding suggests that *Tfec* could have a specific function in splenic macrophages. *Tcf7l2* is associated with differentiation of plasmacytoid dendritic cells (78). Plasmacytoid dendritic cells express *Csf1r* and were found to be 70% reduced in the spleen of *op/op* (CSF1-deficient) mice (79). The alteration of the gene expression profile in the spleen of *Csf1r*-deficient rats is consistent with the immunohistochemistry (IHC) data and indicates almost complete loss of the marginal zone macrophage populations. These cells, and the related Ag capture cells in the subcapsular sinus of lymph nodes, were also depleted in *op/op* mice (47). Genes that were less spleen specific, but are nevertheless *Csf1r* dependent (e.g., those in cluster 13 and 30), may be shared by macrophages of the marginal zone and red pulp or specifically associated with red pulp macrophages.

*Csf1r*^−/−^ rats had no microglia detected by staining for IBA1 in the brain and retina or in flow cytometry profiles of brain digests (Figs. 8D–G, 9A). This observation was strongly supported by the network analysis of gene expression in the four brain regions shown in Fig. 10B. Cluster 10 (Supplemental Table I) provides a list of 107 rat microglia-enriched genes that have been previously published as expressed in microglia from mouse (21, 39, 40, 80, 81) and human (81, 82). Cluster 10 also includes 18 of the top 35 genes downmodulated in the brains of mice treated with a CSF1R kinase inhibitor to deplete microglia (83). As expected, *Csf1r* was almost undetectable in the homozygotes and was the only transcript that was also reproducibly reduced, by 40–50%, in the heterozygous mutants, in all brain regions. Expression of *Cx3cr1*, as well as cathepsin S (*Ctss*), the enzyme required for CX3CL1 cleavage (84), was also almost completely ablated, to the same extent as *Csf1r*. Microglia-associated transcripts are of particular interest given the emerging consensus that these cells are central players in the pathologic condition of Alzheimer disease. Genes including *Csf1r* and others within cluster 10, including *Trem2*, *C1q*, *Tyrobp*, *Abi3*, and *Spi1*, are associated with disease susceptibility (85–87).

Many known macrophage-associated transcripts were less affected by *Csf1r* mutation, including *Cd14*, *Csf2ra*, *Mertk*, *Fcgr2a* (CD32), *Stab1*, *Msr1*, *Marco*, *Gpr84*, *Icam1*, *Clec7a*, and several TLR (*Tlr2,4,8,9,11*) (average 25% less expression; Supplemental Table I). Among macrophage-expressed transcription factors, *Spi1* (PU.1) was reduced by around 75%, but *Irf8*, *Cebpa*, *Cebpb*, *Runx1*, *Tfec*, and *Stat5A*, all of which were readily detectable and are expressed by microglia in the mouse (39, 41), were reduced by <37%. One interpretation is that these transcripts are more highly expressed in the nonmicroglial macrophage population, which is also less *Csf1r* dependent. The CD45^hi^ brain macrophage population in rats were isolated and characterized by Ford et al. (88). They were larger than microglia, with a lower nucleus/cytoplasm ratio. A subset expressed MHC-II; notably, aside from RT1-DMb, the expression of MHC-II genes (RT1-DMa, RT1-Da, RT1-Db1, RT-1DB2, RT1-DOb) was not affected by the *Csf1r* mutation. There was no detectable reduction in lysosome/endosome-associated genes (e.g., *Gpnmb*, *Ctsb*, *Lipa*, *Tcirg1*) that in mice are very strongly enriched in macrophages and microglia compared with total brain (see www.biogps.org). An alternative interpretation is that the loss of microglial endocytic activity is compensated by increased activity in other nonmacrophage cells.

Few impacts on overall brain architecture are shared with the *Csf1r* knockout mouse (27). There was little overt phenotype and specifically no impact on the olfactory bulb, where the mouse mutation had the greatest effect (Fig. 8B). A recent study described a specific microglial population associated with myelination in the brain in the postnatal period (89). These cells were proposed to be the major source of IGF1 required for myelination. The loss of these cells could explain the minor deficiency of myelination and myelination-associated transcripts (Figs. 8C, 9B) observed in the mutant rats. However, in the gene expression profiles of rat brain regions, there was no deficiency in *Igf1* mRNA associated with the *Csf1r* mutation. Similarly, microglia have been considered an important source of brain-derived neutrotrophic factor (BDNF), although conditional microglial depletion did not reduce the total level of *Bdnf* mRNA in the mouse cortex or hippocampus (90). *Bdnf* mRNA was not reduced in the *Csf1r* mutant rat brain. Presumably, the other cellular sources of these trophic factors can compensate for the absence of microglia. It remains to be seen whether the loss of microglia produces the alterations in synaptic proteins and synaptic plasticity observed in mouse microglia depletion studies (90, 91). Microglia in mice have been associated with the outgrowth of dopaminergic neurons, and their depletion produced an imbalance in dopaminergic innervation of the striatum (92). In the striatum of *Csf1r*-deficient rats (Supplemental Table I), we observed a 30–40% increase in mRNA encoding dopamine receptors (*Drd1*, *2*, and *3*), the dopamine transporter *Slc41a3*, and other genes correlated with dopamine receptor expression across brain regions, *Gnal*, *Dgkb*, *Pcp4l1*, *Kcna*, and *Tac1* (93). More subtle brain phenotypes may emerge from further investigation. The advantage of the rat model is that these phenotypes can be studied in adult animals in the absence of gross changes in brain architecture. The flow cytometry analysis and gene expression profiling indicate that there is no compensatory monocyte recruitment or inflammatory cytokine production in the *Csf1r*^−/−^ brain, consistent with the absence of circulating monocytes. Monocyte recruitment is the hallmark of brain pathologic conditions (38, 94) and has been observed in other models of microglial deficiency (40). We suggest that the impact of the loss of the neuroprotective functions of microglia in the *Csf1r*^−/−^ rat may be mitigated by the absence of monocytes.

The human disease ALSP [formerly known as hereditary diffuse leukoencephalopathy with spheroids (95)] is associated with mutations affecting conserved amino acids in the kinase domain of CSF1R (96), which are likely to have a dominant-negative impact on signaling by forming inactive heterodimers with the wild type receptor (8). Fifty-eight different mutations have been described in ALSP patients (7), and within the human exome database (exac.broadinstitute.org), there are a further 38 variants, each identified only as a singleton, that affect amino acids conserved from humans to chickens. One report claimed that the disease could be associated with haploinsufficiency arising from a frame-shift mutation (7), but their data indicate >50% loss of CSF1R protein in the brains of these patients. Chitu and colleagues (97) have characterized *Csf1r*^+/−^ inbred mice as a model for ALSP. Like these authors, we found that there is no dosage compensation for loss of one *Csf1r* allele, which is surprising because CSF1 signaling regulates *Csf1r* mRNA levels (98). However, despite the lack of dosage compensation, we have found no significant impact of the *Csf1r* heterozygous mutation on gene expression in the adult brain (Supplemental Table I). We have maintained heterozygous mutant rats for more than 12 months and observed no overt phenotype. Accordingly, the impact of dominant-acting mutations in patients is likely to depend in part upon the level of expression of the other allele and on the activity of the compensatory mechanisms that permit relatively normal brain function when microglial differentiation is compromised.

In overview, analysis of *Csf1r*^−/−^ rats highlights selective impacts of the mutation on macrophage populations and clear differences in the cell populations affected and the pleiotropic consequences compared with mutant mice. The effect of *Csf1* mutation in mice on macrophages and osteoclast populations is gradually corrected with age. The macrophage phenotype can be corrected with exogenous GM-CSF (CSF2), but endogenous CSF2 is not required (99). Conversely, mutation of *Flt1* prevents the age-dependent development of osteoclasts (100). As noted above, one major difference between rats and mice is that rat macrophages themselves express high levels of *Csf1* mRNA. This explains the loss of *Csf1* mRNA in the spleen of *Csf1r* mutant rats. In the brain of rats, *Csf1* and *Il34* were both highly expressed and not notably region specific. The *Csf1r* mutation did not reduce the level of expression, suggesting that microglia are not the major source. We suggest that differences between the rodent species derive from differences in expression or function of alternative growth factors that can compensate for the lack of *Csf1r.* Although female *Csf1r* mutant rats appear infertile, hormone-dependent expression of CSF2 and other factors by the uterus (101) might also mitigate the impact of the *Csf1r* mutation on macrophage numbers and function in females relative to males.

The increased longevity and plethora of phenotypes found in *Csf1r*^−/−^ rats provide a unique model for studying the role of CSF1R during development and adulthood, the functions of microglia, and other tissue macrophage populations and the molecular basis for adult onset disease associated with human *CSF1R* mutations. Many of the macrophage phenotypes are shared between mice and rats, so the differences mainly lie in the relative redundancy of macrophages in developmental processes in the two species.

## Supplementary Material

Data Supplement
