# Correction: Pleiotropic Impacts of Macrophage and Microglial Deficiency on Development in Rats with Targeted Mutation of the *Csf1r* Locus

**DOI:** 10.4049/jimmunol.1900420

**Published:** 2019-04-26

**Authors:** Clare Pridans, Anna Raper, Gemma M. Davis, Joana Alves, Kristin A. Sauter, Lucas Lefevre, Tim Regan, Stephen Meek, Linda Sutherland, Alison J. Thomson, Sara Clohisey, Stephen J. Bush, Rocío Rojo, Zofia M. Lisowski, Robert Wallace, Kathleen Grabert, Kyle R. Upton, Yi Ting Tsai, Deborah Brown, Lee B. Smith, Kim M. Summers, Neil A. Mabbott, Pedro Piccardo, Michael T. Cheeseman, Tom Burdon, David A. Hume

Pridans, C., A. Raper, G. M. Davis, J. Alves, K. A. Sauter, L. Lefevre, T. Regan, S. Meek, L. Sutherland, A. J. Thomson, S. Clohisey, S. J. Bush, R. Rojo, Z. M. Lisowski, R. Wallace, K. Grabert, K. R. Upton, Y. T. Tsai, D. Brown, L. B. Smith, K. M. Summers, N. A. Mabbott, P. Piccardo, M. T. Cheeseman, T. Burdon, and D. A. Hume. 2018. Pleiotropic impacts of macrophage and microglial deficiency on development in rats with targeted mutation of the *Csf1r* locus. *J. Immunol.* 201: 2683–2699.

In the analysis of gene expression in the brain, the sample labels for the pituitary gland and olfactory bulb were reversed. As a consequence, the clusters in Fig. 10B were mislabeled. A corrected version of Fig. 10 is shown below, along with the corrected figure legend. These corrections have been made to the online version of the article, which now differs from the print version as originally published. A corrected version of Supplemental Table I has also been published online. The current online supplemental material therefore differs from what was originally published online. The relevant text in the *Results* section under the heading “*Network analysis of gene expression in the brain of Csf1r-deficient rats*” now reads as follows:

**FIGURE 10.**
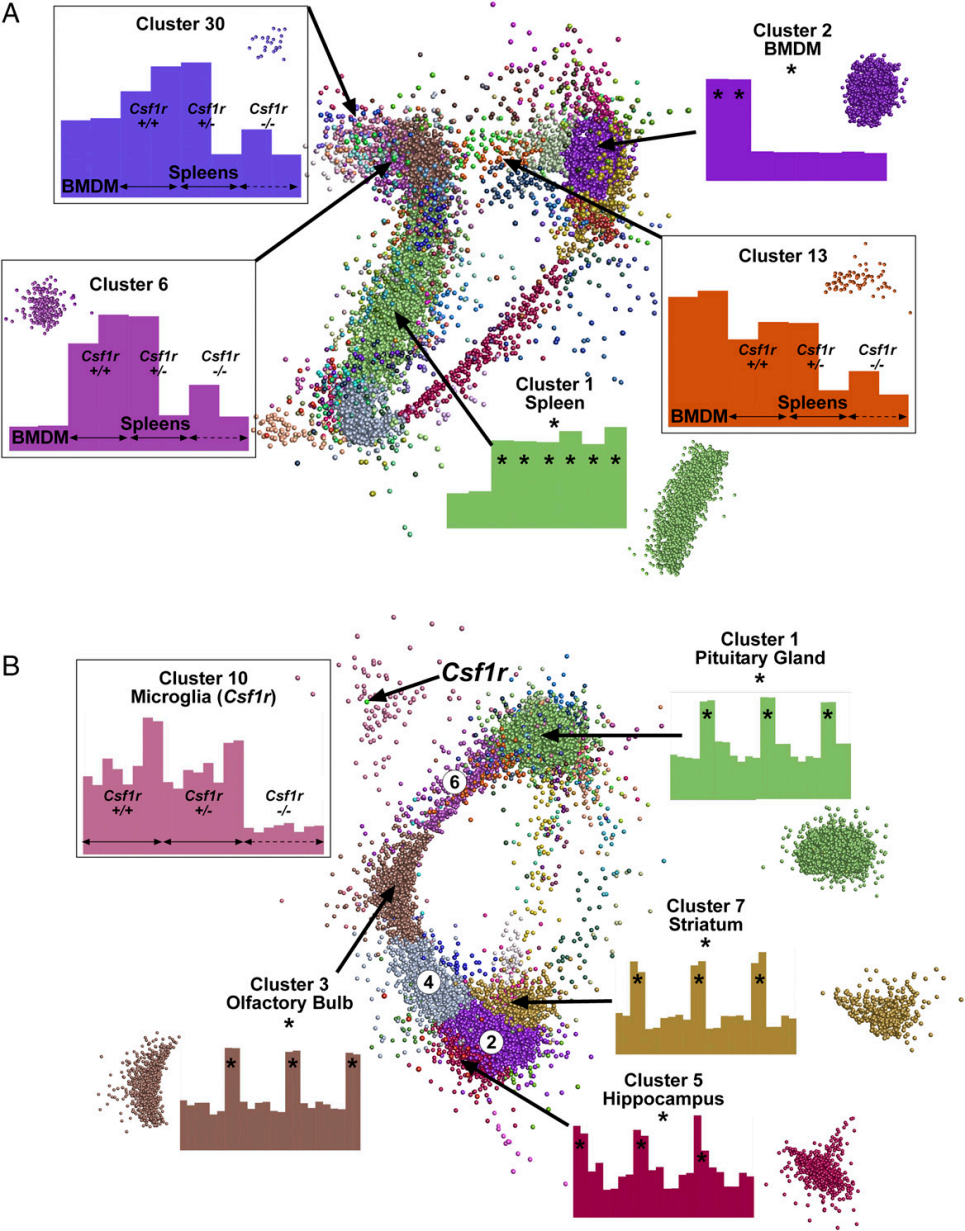
Network analysis of gene expression in the spleen and brains of *Csf1r* deficient rats. RMA-normalized microarray data from Supplemental Table I was analyzed with Graphia Pro. Edges have been removed for ease of visualization. Nodes allocated to the same cluster are the same color. Histograms show the averaged expression patterns of all genes in the cluster. Boxed clusters refer to genes affected by loss of *Csf1r*. Unboxed clusters refer to genes that are tissue specific (*). (**A**) Key clusters from spleen. All known genes in which no sample reached an intensity of 20 were excluded. Analysis was performed at a Pearson correlation coefficient ≥0.95 (12,305 nodes making 1,746,925 edges). Clustering was performed at an inflation of 2.0 with a minimum cluster size of 10. (**B**) Key clusters from brain. All known genes in which no sample reached an intensity of 20 were excluded. Analysis was performed at a Pearson correlation coefficient ≥0.85 (11,833 nodes making 3,617,804 edges). Clustering was performed at an inflation of 2.0 with a minimum cluster size of 10. Three clusters (circled numbers) shared gene expression with multiple brain regions: cluster 2 (striatum and hippocampus), cluster 4 (olfactory bulb, striatum, and hippocampus), and cluster 6 (olfactory bulb and pituitary gland). Histograms for these clusters are shown in Supplemental Table I.

Fig. 10B shows the network graph for the combined analysis of the four brain regions; gene lists are provided in Supplemental Table I. The largest cluster was cluster 1 (4283 genes), containing pituitary gland–associated genes. Other region-specific clusters were cluster 3 (olfactory bulb; 878 genes), cluster 5 (hippocampus; 513 genes), and cluster 7 (striatum; 384 genes). The second largest cluster (cluster 2; 1253 genes) contained genes that were more highly expressed in both hippocampus and striatum. Two other main clusters also shared highly expressed genes between tissues: cluster 4 (olfactory bulb, striatum, and hippocampus; 757 genes) and cluster 6 (pituitary gland and olfactory bulb; 471 genes). None of these clusters showed any evidence of genotype association.

