# Supplementary material for: Pleiotropic Impacts of Macrophage and Microglial Deficiency on Development in Rats with Targeted Mutation of the Csf1r Locus
Source: J Immunol. 2018 Sep 24;201(9):2683–99. doi: 10.4049/jimmunol.1701783 (PMC6196293; doi:10.4049/jimmunol.1701783)
Supplement: Data Supplement [file JI_1701783.zip › JI_1701783_Supplemental_Figures_1.pdf]

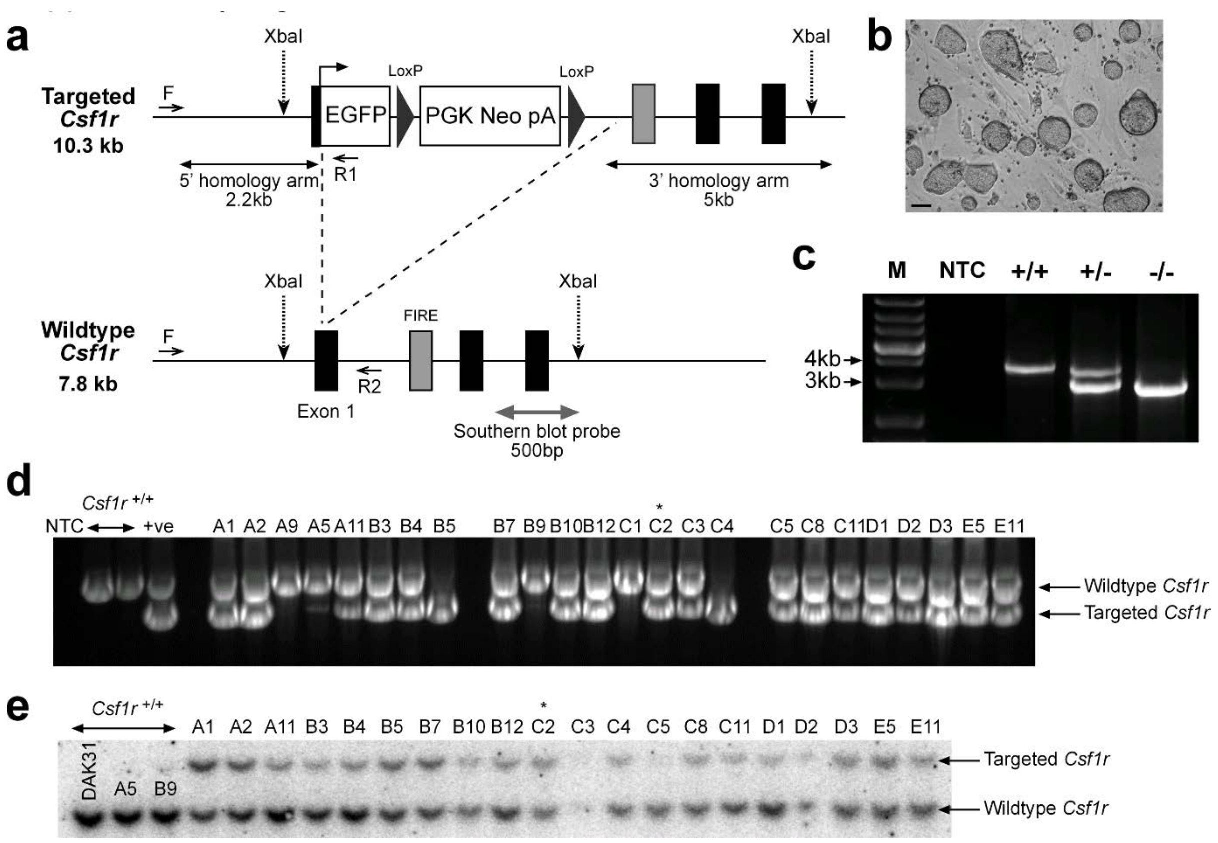

Supplementary Figure 1. **Rat *Csf1r* targeting strategy and screening**

(a) Schematic of the wildtype and targeted rat *Csf1r* alleles showing the location of PCR genotyping primers (F, R1 and R2), XbaI restriction enzymes sites used in Southern blot analysis and the location of the 3’ Southern blot probe. (b) Rat ESC clone DAK31-C2 used to generate the colony. Bar = 50µm. (c) Genotyping PCR. M = marker, NTC = no template control. (d) Genomic DNA (gDNA) from Geneticin resistant ESC clones was isolated to screen the 5’end of the targeted allele via PCR. (e) gDNA from ESC clones were digested with XbaI and used in Southern blot analysis to screen the 3’ end of the targeted allele.

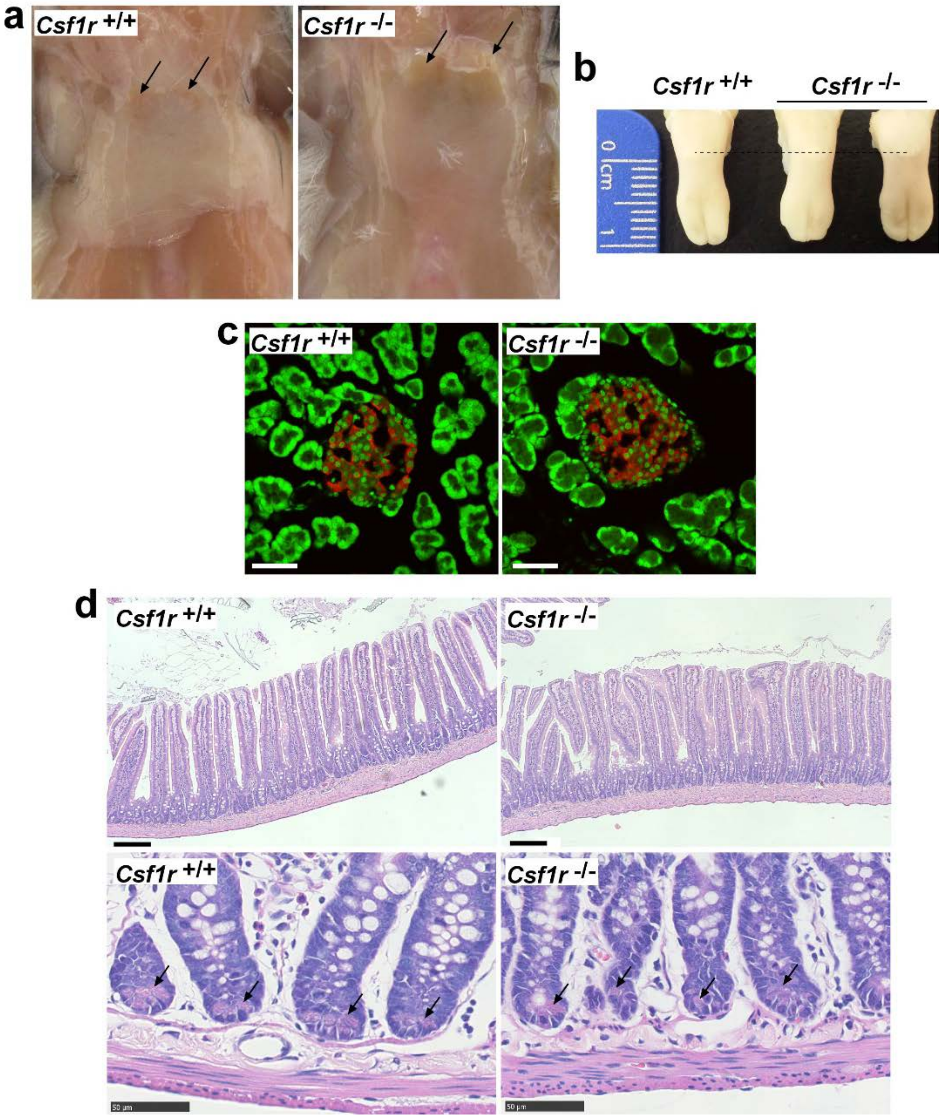

Supplementary Figure 2 – Further phenotypes of *Csf1r* deficient rats

(a) Skin was removed from the dorsum of 4.5 week old rats following euthanasia. Arrows point to interscapular brown adipose tissue. Image is representative of males and females. (b) Formalin-fixed oral tongues from 4.5 week old rats (dorsal surface). Dotted line highlights the intermolar eminence. Image is representative of males and females. (c) PFA-fixed cryosections of pancreata were immunostained with anti-insulin antibody (red) and counterstained with Cytogreen. n=2 per genotype, 5 islets of Langerhans per rat. Bar = 50µm. (d) Formalin-fixed paraffin-embedded sections of small intestine were stained with haematoxylin and eosin. Images are representative of 10 adult rats per genotype. Arrows point to Paneth cells. Bar = 100µm (upper panel) and 50µm (lower).

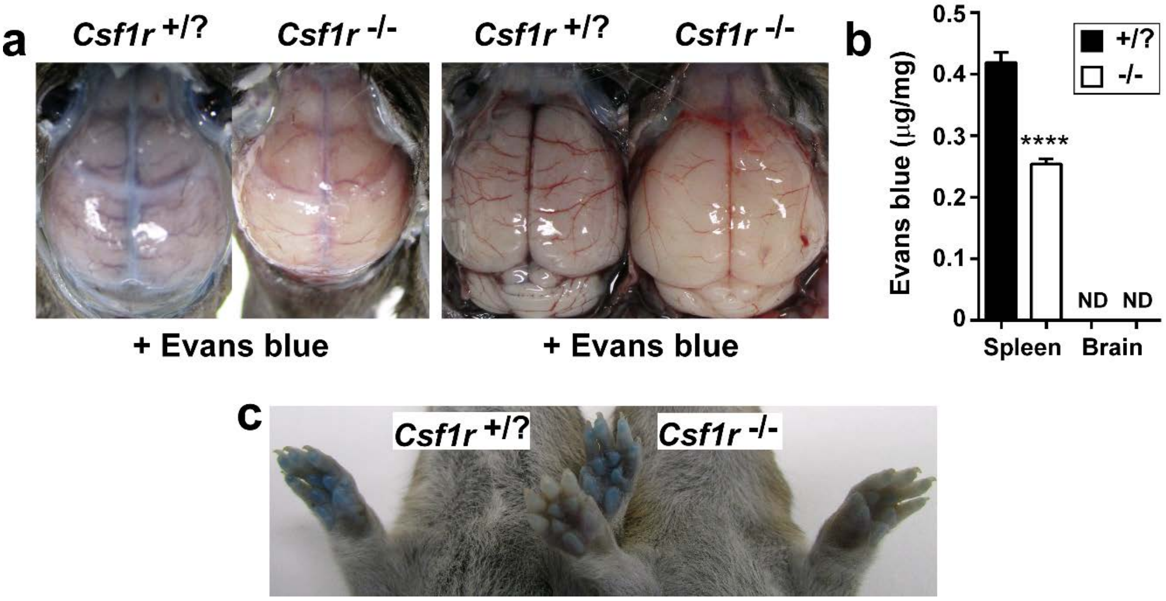

Supplementary Figure 3 – Blood brain barrier permeability experiments

Rats at postnatal day 17 were injected with Evans Blue stain and euthanized 3 hours later. (a) Skulls remained intact (left) or were removed (right). (b) Formamide was used to extract Evans blue dye from dried organs and concentrations determined by spectrophotometry using a standard curve. Graph shows mean + SEM. ND = not detected. Significance compared to littermates (+/?) is indicated by ****P < 0.0001 using a t-test. (c) Photograph of rats post-euthanasia. Images are representative of 6 littermates (*Csf1r*$^{+/?}$), 4 *Csf1r*$^{-/-}$ rats and 2 repeat experiments.
